# Isolation of potentially novel species expands the genomic and functional diversity of Lachnospiraceae

**DOI:** 10.1002/imt2.174

**Published:** 2024-02-13

**Authors:** Xiaoqian Lin, Tongyuan Hu, Zhinan Wu, Lingne Li, Yuhao Wang, Dingyang Wen, Xudong Liu, Wenxi Li, Hewei Liang, Xin Jin, Xun Xu, Jian Wang, Huanming Yang, Karsten Kristiansen, Liang Xiao, Yuanqiang Zou

**Affiliations:** ^1^ BGI Research Shenzhen China; ^2^ School of Bioscience and Biotechnology South China University of Technology Guangzhou China; ^3^ College of Life Sciences University of Chinese Academy of Sciences Beijing China; ^4^ James D. Watson Institute of Genome Sciences Hangzhou China; ^5^ Laboratory of Genomics and Molecular Biomedicine University of Copenhagen Copenhagen Denmark; ^6^ Shenzhen Engineering Laboratory of Detection and Intervention of human intestinal microbiome, BGI‐Shenzhen Shenzhen China

**Keywords:** genomic analysis, Lachnospiraceae, next‐generation probiotics, probiotic functional predictions

## Abstract

The Lachnospiraceae family holds promise as a source of next‐generation probiotics, yet a comprehensive delineation of its diversity is lacking, hampering the identification of suitable strains for future applications. To address this knowledge gap, we conducted an in‐depth genomic and functional analysis of 1868 high‐quality genomes, combining data from public databases with our new isolates. This data set represented 387 colonization‐selective species‐level clusters, of which eight genera represented multilineage clusters. Pan‐genome analysis, single‐nucleotide polymorphism (SNP) identification, and probiotic functional predictions revealed that species taxonomy, habitats, and geography together shape the functional diversity of Lachnospiraceae. Moreover, analyses of associations with atherosclerotic cardiovascular disease (ACVD) and inflammatory bowel disease (IBD) indicated that several strains of potentially novel Lachnospiraceae species possess the capacity to reduce the abundance of opportunistic pathogens, thereby imparting potential health benefits. Our findings shed light on the untapped potential of novel species enabling knowledge‐based selection of strains for the development of next‐generation probiotics holding promise for improving human health and disease management.

## Highlights

- Comprehensive genome analysis reveals underestimated species diversity and distinct functions of Lachnospiraceae.
- Cataloging 1.5 M genes highlights Lachnospiraceae's significant role in the human gut microbiota, with potential novel species contributing.
- Lachnospiraceae demonstrates a vast potential for synthesizing short‐chain fatty acid (SCFA), producing secondary metabolites, and forming spores, which is promising for future studies and applications.
- The abundance of Lachnospiraceae correlates inversely with diseases like atherosclerotic cardiovascular disease (ACVD) and inflammatory bowel disease (IBD), indicating a potential protective role.

## Graphical abstract

We present a comprehensive exploration of Lachnospiraceae, leveraging an extensive cultured‐based genome collection. Phylogenetic analysis reveals previously underestimated taxonomic diversity. A thorough gene set analysis, comprising a 1.5 M gene catalog, sheds light on the functional richness of Lachnospiraceae. Notably, exploration of short‐chain fatty acid synthesis pathways highlights their synthetic potential, with strains displaying distinct capacities for butyrate and propionate production. Disease association analysis establishes correlations between Lachnospiraceae strains and atherosclerotic cardiovascular disease and inflammatory bowel disease, offering valuable insights into their potential roles in human health and disease.

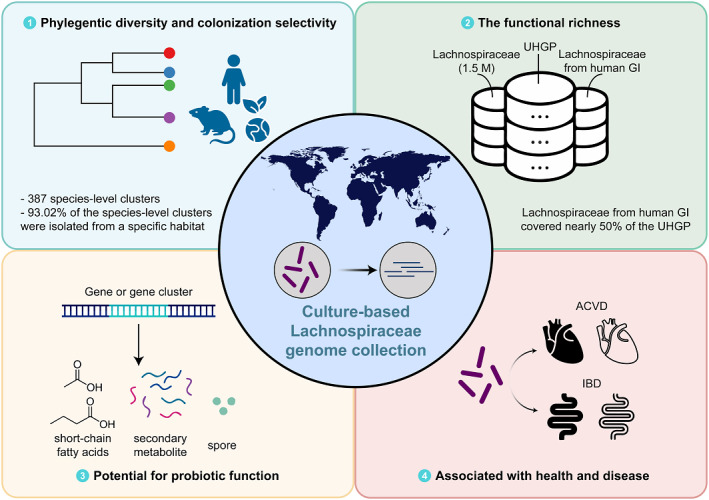

## INTRODUCTION

Members of Lachnospiraceae, a family within the Bacillota phylum comprising several strictly anaerobic genera, are abundant in the intestines of mammals, particularly humans and ruminants, and members of this family also colonize the environment [1]. Previous studies have revealed a significant association between members of the Lachnospiraceae family and several diseases based on metagenomic data [2, 3, 4, 5]. In addition, *Roseburia* spp. were found to be significantly reduced in individuals with atherosclerotic cardiovascular disease (ACVD) [6, 7]. Many species within Lachnospiraceae contribute important functions, such as bile acid conversion, short‐chain fatty acid production, and antibiotic production in the human gastrointestinal tract [8, 9, 10], and several members have been reported to be associated with beneficial effects on human health. Thus, oral intake of *Anaerobutyricum soehngenii* has been reported to improve insulin sensitivity in individuals with metabolic syndrome [11], and *Anaerobutyricum hallii* has been reported to improve postprandial blood glucose control in patients with type 2 diabetes [12]. Accordingly, bacteria of the Lachnospiraceae family seem to hold promise as interesting next‐generation probiotic candidates.

However, studies have also indicated that some strains of Lachnospiraceae may promote disease development. *Catonella morbi* ATCC 51271 isolated from the oral cavity is thought to be associated with periodontitis [13], *Anaerostipes hadrus* BPB5 has been shown to aggravate colitis in dextran sodium sulfate‐treated mice [14], and *Eisenbergiella tayi* isolated from human blood even acts as an opportunistic pathogen [15]. Therefore, it is extremely important to select the right species or strain for future preclinical research. Previous studies analyzing isolated strains of five genera in the Lachnospiraceae family have revealed high diversity between human‐derived isolates [9], but Lachnospiraceae contains at least 58 genera and 122 valid‐and‐correct‐name species in The List of Prokaryotic names with Standing in Nomenclature (LPSN, https://lpsn.dsmz.de/, up to July 2021) [16]. Thus, although a large number of studies have examined the impact of Lachnospiraceae on host health, most taxa of interest lack species‐level taxonomy, implying that there is a large number of potentially new species, warranting further studies on the genomic diversity of Lachnospiraceae.

In our previous study expanding the bacterial collection of the Cultivated Genome Reference (termed CGR2) [17], we cultured 756 Lachnospiraceae strains from the feces of healthy Chinese adults and released high‐quality genomes. By collecting available culture‐based genome data from public databases and combining these data with the genomes of CGR2, we constructed a collection comprising 1868 high‐quality genomes belonging to Lachnospiraceae. These genomes revealed a significantly increased taxonomic diversity in the Lachnospiraceae family, and the potentially new species greatly expanded the existing profiles of genes and functions. In addition, the comprehensive Lachnospiraceae cultivated genome collection improved the resolution of disease‐related markers and provided a basis for selecting strains with potentially beneficial effects on human health.

## RESULTS

### The expanded Cultivated Genome Reference increases the taxonomic diversity of Lachnospiraceae

**Figure 1:**
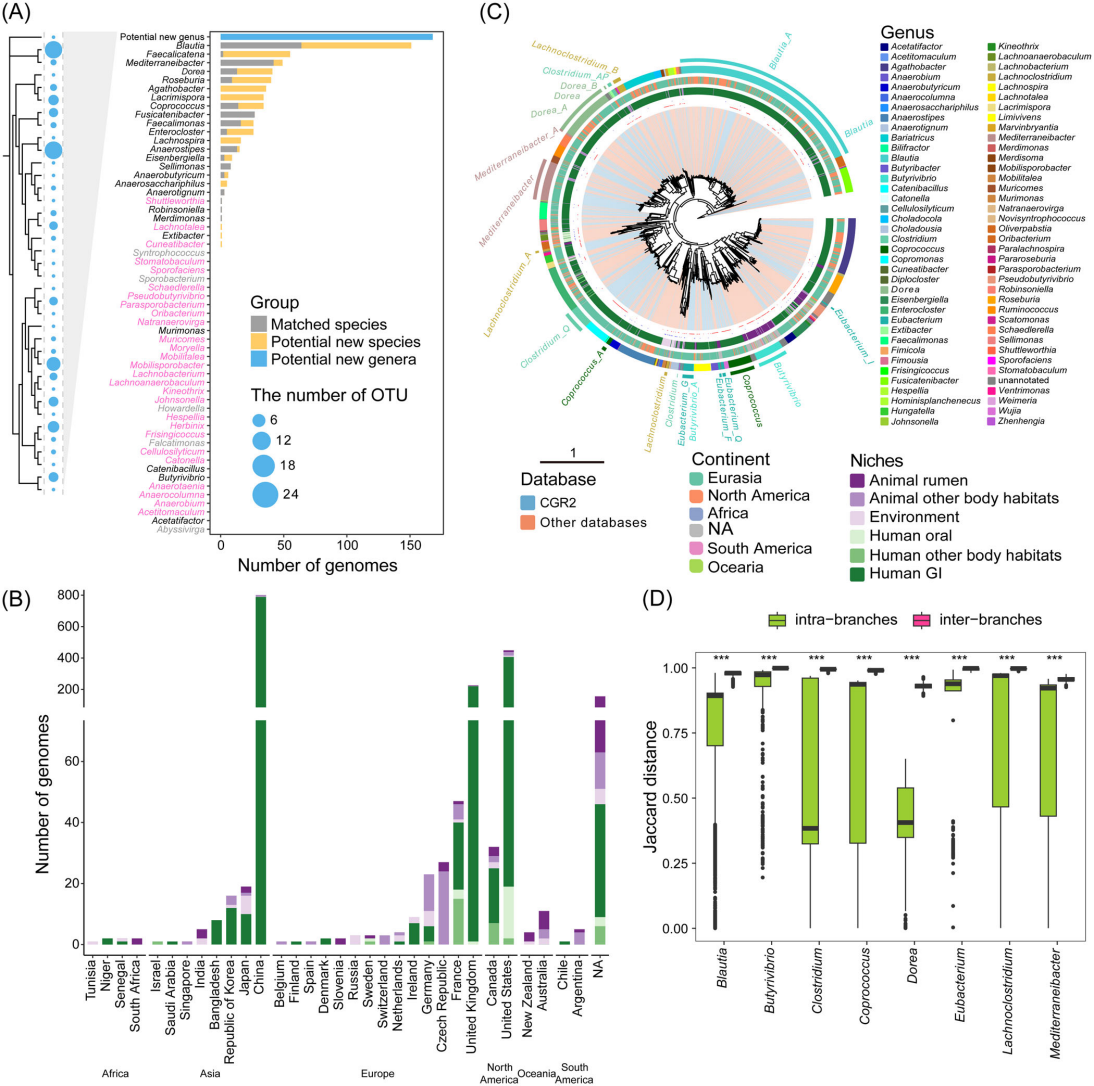
The collection of 1868 Lachnospiraceae cultured genomes. (A) Contribution of 756 newly isolated Lachnospiraceae genomes to the existing valid name genera. Genera represented by the human gut cultured genomes are marked in black, genera without human intestinal culture representation but with genomes from other niches are marked in pink, and genera without any cultured genome are marked in gray. (B) Geographical and niche distribution of the number of genomes retrieved. (C) Phylogenetic tree of the 1868 isolated genomes. The tree was produced from concatenated protein sequences using PhyloPhlAn 3. The clades are colored according to the genome source database (The expanded Cultivated Genome Reference [CGR2] or download). Potentially novel species and type strains are marked in the first layer with red and blue dots, respectively. The second and third layers represent the niches and continents where the genomes were isolated, respectively. The GTDB genus annotation is marked at the last layer and text‐labeled for those with multiple lineages. (D) The Jaccard distance distributions of eight multilineage genera within and between branches. ***p < 0.001 as defined by the Wilcoxon test.

We obtained 756 high‐quality Lachnospiraceae genomes from the expanded Cultivated Genome Reference (CGR2) [17]. To evaluate the novelty of these genomes, we retrieved 58 genera and 122 valid‐named species from LPSN and downloaded their 16S ribosomal RNA (rRNA) gene sequences as a reference. We found that 47.88% of the newly cultured genomes in CGR2 were potentially novel species, and 22.22% corresponded to potentially novel genera, using similarities of 98.7% and 94.5% as the species and genus demarcation [18], respectively. In addition, the 16S rRNA gene sequences of the potentially novel genera were clustered into 37 genus‐level operational taxonomic units (OTUs) and 64 species‐level OTUs. Notably, the genomes from CGR2 not only covered most genera of the Lachnospiraceae family identified in the human gut microbiota but also added three potentially new genera that had not been isolated previously from the human gut (Figure 1A). Together, these results increased the taxonomic diversity of Lachnospiraceae, warranting further studies to fully explore the diversity of Lachnospiraceae.

Next, we collected isolated genomes of Lachnospiraceae from the NCBI (939), IMG (5), and the Unified Human Gastrointestinal Genome (UHGG) collection (190) (as of August 2021). CheckM quality control resulted in 1868 high‐quality genomes, including 756 genomes from CGR2. Strains were isolated from multiple sources, including humans, animals, and the environment from a total of 32 countries across six continents (Africa, Asia, Europe, North America, Oceania, and South America), which highlights the prevalence of Lachnospiraceae in different regions and countries (Figure 1B and Table S1). This notion is in accordance with earlier studies on the prevalence and abundance of Lachnospiraceae in metagenomic data, indicating that members of the Lachnospiraceae family are common in samples of the mammalian gastrointestinal tract and the environment [1].

### Phylogenetic analysis reveals phyletic diversity and colonization selectivity

Based on the 95% average nucleotide identity (ANI) threshold, all 1868 genomes were clustered into 387 species‐level clusters, exceeding by a factor of three the number of previously annotated species (Table S1 and S2). Digital DNA–DNA hybridization (dDDH) is another bioinformatics technique used to estimate the genetic relatedness or similarity between two bacterial genomes. The dDDH values within clusters and between clusters support the current delineation of species‐level clusters in our study (Figure S1). A number of genera, including *Blautia*, *Copromonas*, *Butyrivibrio*, *Coprococcus*, and *Pseudobutyrivibrio*, harbored a large number of potentially new species (Figure 1C and Table S1). Among eight genera, *Blautia*, *Butyrivibrio*, *Clostridium*, *Coprococcus*, *Dorea*, *Eubacterium*, *Lachnoclostridium*, and *Mediterraneibacter*, we observed that at least two distinct branches were evident in the phylogenetic tree (Figure 1C), with the genetic composition confirming the divergence between these branches (Figure 1D). Notably, interbranches had quite high and more narrowly distributed Jaccard values, with median values ranging from 93.03% to 99.86% (Figure 1D). These values were significantly higher than those observed within intrabranches. This indicates a greater genetic variability among interbranches, suggesting a potential rationale for their appropriate grouping.

The ANI and 16S rRNA gene sequences similarity are two commonly used strategies for species demarcation in prokaryotes, but these two methods may create biases. Therefore, we investigated differences in species demarcation between the two methods. Using 109 sequenced type strain genomes and 16S rRNA gene downloaded sequences as references, we found 26 species‐level clusters with conserved 16S rRNA gene sequences, but highly diverse genomes, which were annotated as new species or multiple phyletic lineages by the GTDB (Figure S2). Conversely, the seven species‐level clusters exhibited low 16S rRNA gene similarity to the type strain (Figure S2). Additionally, we blasted the 16S rRNA gene sequences predicted by the type strain genome against the downloaded sequences, arriving at the same result. Therefore, a thorough analysis of the possible contamination of the 16S rRNA gene sequences of the seven species‐level clusters in GTDB is warranted.

In general, the genomes represented species isolated from humans, animals, and the environment, corroborating the findings of previous studies [1]. Based on the culture method, we can trace the origin at the species or even the strain level. We found that 30 out of 75 genera were isolated from different body habitats, mainly from humans and animals, whereas 93.02% of the species‐level clusters were isolated from a specific habitat, indicating the selective colonization of individual members of the Lachnospiraceae family (Figure S3). Statistical analysis showed that both genera and species were correlated with habitat (*χ* ^2^ test, *p* < 0.01).

### Sets of genes and proteins in Lachnospiraceae genomes

**Figure 2:**
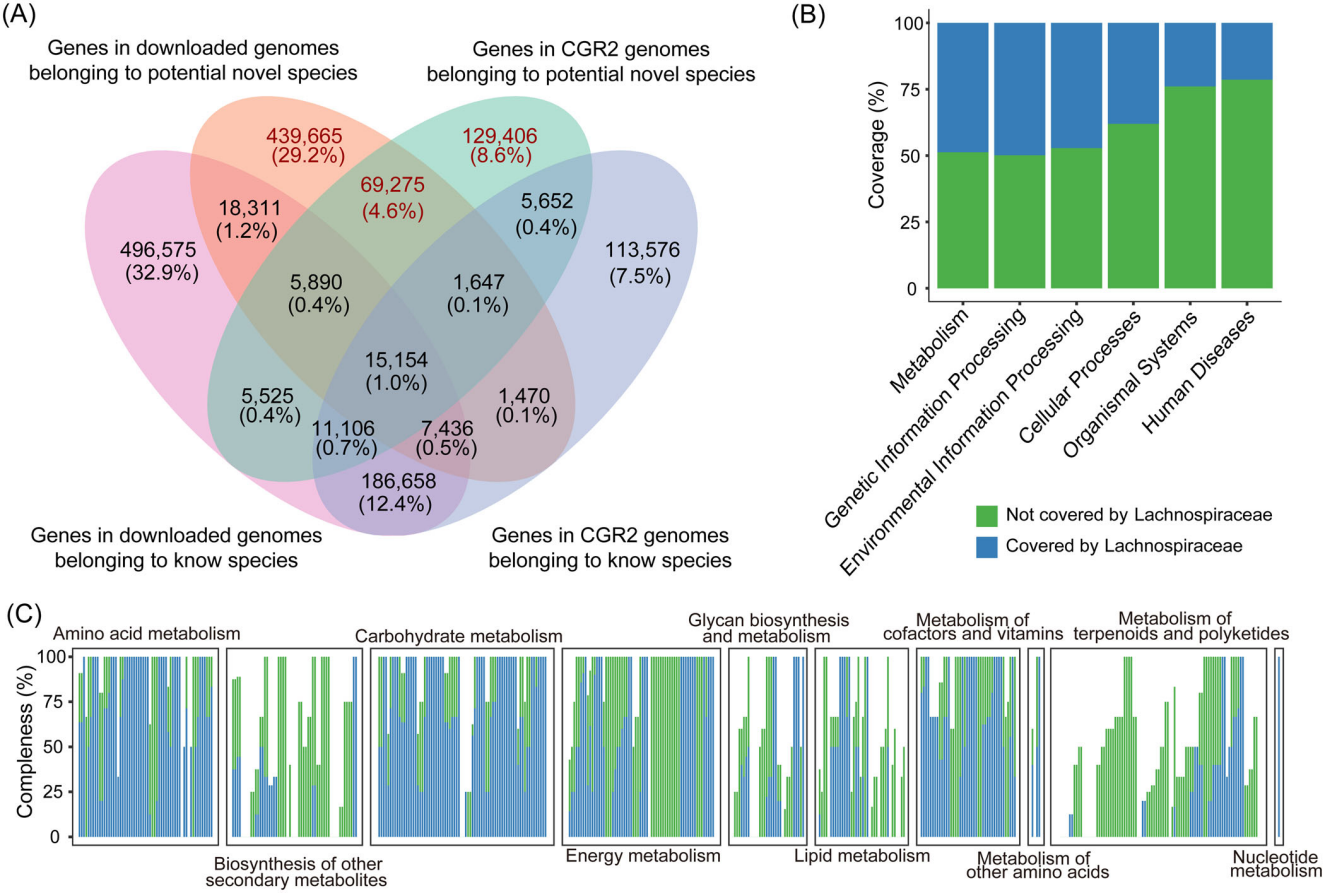
New insights into the genes of Lachnospiraceae*.* (A) The 1.5 M isolated Lachnospiraceae gene catalog, with parts unique to the genomes of potentially new species highlighted in red. (B) The percentage of human gut Lachnospiraceae genes contributing to the human gut microbiota function. (C) The completeness of each metabolic functional module. A bar represents a module, and all modules can be divided into 10 categories according to their functions.

To establish functional profiles, we constructed a gene catalog based on 1868 genomes. The results illustrated that the new genomes from the cultured species from CGR2 expanded the Lachnospiraceae gene catalog to 1.5 M (Figure 2A). We investigated the contribution of potentially novel species to the gene catalog, which did not surprisingly reveal that these hitherto unknown species contributed 42.34% of the genes, as highlighted in red in Figure 2A. Furthermore, genes encoding methyl‐accepting chemotaxis protein, endoglucanase, peptide/nickel transport system substrate‐binding protein, and flagellin were enriched in the potentially novel species, indicating that these species may provide new insights into glucose metabolism, motility, and other aspects of members of the Lachnospiraceae family (Figure S4B).

Turning our focus toward protein sequences encoded by these genes, we set out to construct a protein sequence catalog. Similar to the gene catalog, we obtained a 1.4 M protein catalog (Figure S4A). It is noticeable that more than half (55.52%) of the catalog are hypothetical proteins based on annotations in the Prokka database.

Our quest to determine the influence of Lachnospiraceae family members on the functions of the human gut microbiota led us to extract protein sequences predicted from genomes sourced from the human gastrointestinal tract. Subsequently, we analyzed these sequences using the Unified Human Gastrointestinal Protein (UHGP) catalog, renowned as the most comprehensive repository of proteins from the human gut microbiome. The results showed that members of the Lachnospiraceae family isolated from the human gastrointestinal tract covered nearly 50% of the functions of the human intestinal microbiota, including metabolism, genetic information Processing, and environmental information processing (Figure 2B).

Given the pivotal role played by gut microbes in host nutrition and metabolism, our analyses focused on the metabolic functions of Lachnospiraceae family members originating from the human gut. We demonstrated that these members not only have a great capacity for carbohydrate metabolism, fatty acid synthesis, and degradation but also participate in branched‐chain amino acid biosynthesis, purine and urea metabolism, and folate biosynthesis, which are important for the regulation of host physiology (Figure 2C and Figure S4C). In addition, members of the human intestinal tract Lachnospiraceae family contributed 59 unique KEGG Orthologies (KOs) (Figure S4D), mainly involved in synthetic and metabolic functions.

### Pan‐genome analysis reveals the ecological diversity of representatives of Lachnospiraceae

The pan‐genome represents the entire set of genes from all species/strains within a clade, thereby characterizing the diversity between genomes and providing important insights into the evolutionary origin and niche adaptation. Whole‐genome sequencing of isolates has laid the foundation for identifying core and unique genes between closely related strains.

**Figure 3:**
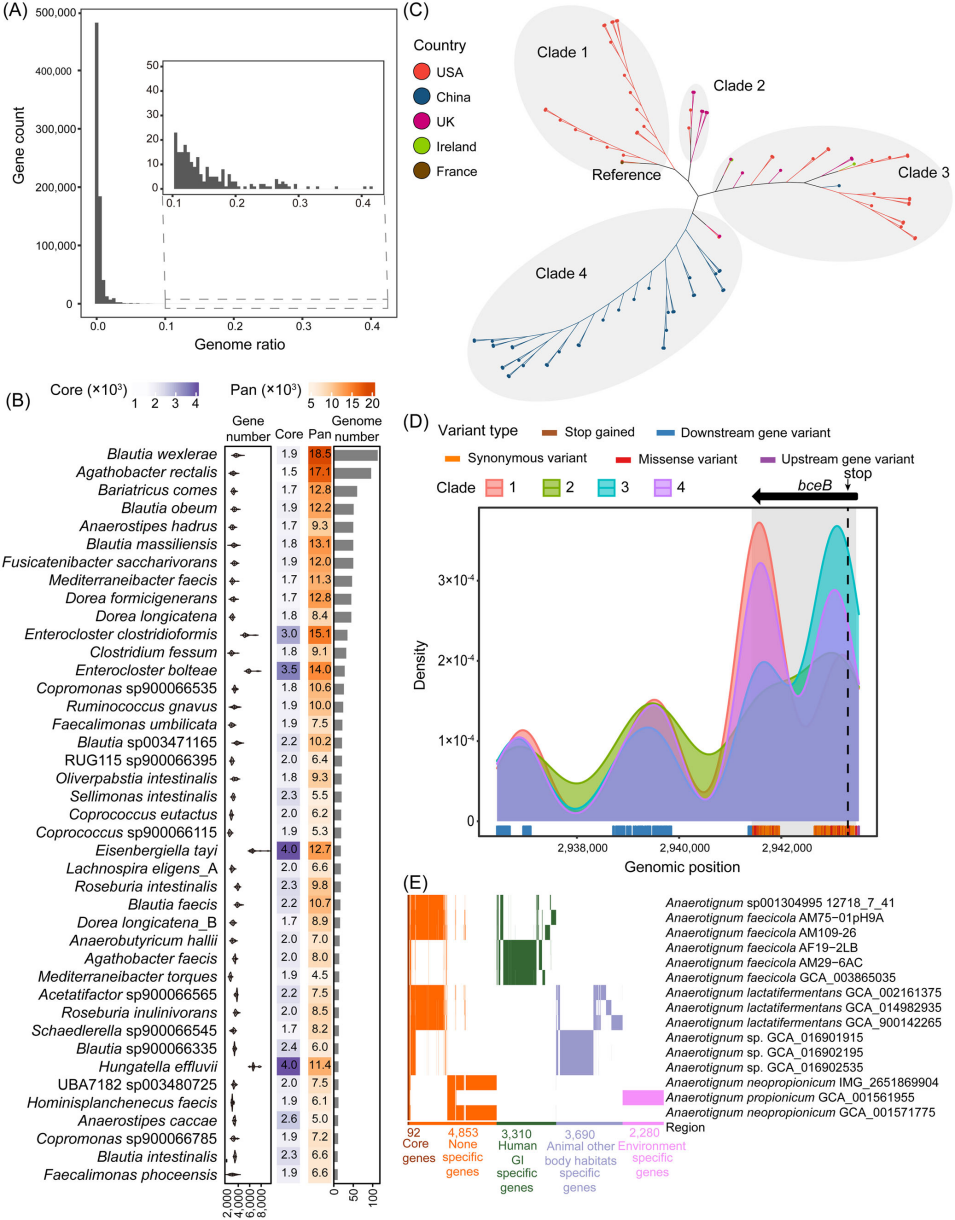
Pan‐genomic diversity of Lachnospiraceae. (A) The number of genes shared between different genomes. The X‐axis is the proportion of genomes containing common genes in all genomes. (B) Core genome, pan‐genome, genome number, and gene number statistics for 41 species with more than 10 genomes sorted by the number of genomes. (C) Phylogenetic unrooted tree constructed based on single‐nucleotide polymorphisms (SNPs). The tree was produced by Parsnp. Nodes are colored according to the isolation country. Clades are divided based on genetic distance. (D) The density of variations frequency at different genome positions of the four clades. The genomic position is colored according to the mutation type. (E) The presence or absence of genes in the *Anaerotignum* genus. Genes that are only present in the genome of a specific niche are defined as specific genes.

First, by constructing a family‐level pan‐genome using all the genomes of the Lachnospiraceae family, we surprisingly found that the most prevalent genes were shared by only 41.54% of the genomes and almost 99.99% of the genes were distributed in only a few genomes, which were defined as Cloud Genes [19] (Figure 3A). We analyzed the pan‐genome and core‐genome sizes of the genera and species, including at least 10 independent conspecific genomes. At the genus level, the sizes of the core and pan‐genomes were positively correlated with the number of genomes and clusters. The range of pan‐genomes varied up to 10‐fold, while the range of core‐genomes varied by more than 1000‐fold (Figure S5A). At the species level, *Hungatella effluvii*, *Eisenbergiella tayi*, *Enterocloster boltteae*, and *Enterocloster clostridioformis* have a larger number of genes and thus larger core and pan‐genome sizes. Additionally, the top 10 species with a large number of genomes have smaller core genomes and larger pan‐genomes, indicating a more diverse genetic composition of the genome (Figure 3B).

*Blautia wexierae* possesses a large pan‐genome and a relatively small core‐genome; however, it lacks a complete reference genome necessary for single nucleotide polymorphism (SNP) analysis. Therefore, we opted for *Agathobacter rectalis*, which has the second‐largest pan‐genome and a complete reference genome. We further conducted SNP analysis of 96 genomes isolated from five countries, using the complete genome GCA_000020605 isolated from France as the reference. We detected 95173 variants in intragenic regions, of which 19357 were missense variants. The SNP phylogenetic tree showed four clades related to geographic location (Figure 3C). The closest to the reference is Clade 1, composed of genomes from the United States, followed by Clade 2, mainly dominated by UK genomes, clade 3, composed of genomes from multiple countries, and Clade 4, mainly consisting of Chinese genomes (Figure 3C). After annotating variants located in intragenic regions, we focused on *bceB*, which encodes the ABC transporter complex BceAB involved in bacitracin export and harbors a large number of missense variants and one highly impacting variant (Figure S6A). The four clades showed different *bceB* gene variation patterns. Clade 4 had the highest variation associated with *bceB*, while Clade 2 had the lowest (Figure S6B). The frequent variations in Clade 1 were at the rear end of the gene, while variations in Clade 3 were at the front end of the gene. In addition, all four clades had one variation that caused premature termination of translation, especially Clade 3, in which variation was identified in 82.14% of the genomes (Figure 3D). The ABC transporter BceAB mediates resistance to antimicrobial peptides such as lantibiotics, bacitracin, and β‐lactam antibiotics. This result suggests that *Agathobacter rectalis* exhibits varying degrees of resistance loss.

To investigate the impact of potentially new species on intragenus diversity, we reconstructed a pan‐genome cumulative curve for five genera (Figure S5B). The addition of a large number of potentially new species provided a larger pan‐genome. Compared with known species, new species have a broader functional potential. For example, the pan‐genome of *Butyrivibrio* was more than tripled compared with that of previously known species (Figure S5C).

Investigation of the core and unique genes of *Anaerotignum*, a genus present in the gastrointestinal (GI) tract of humans and animals, and in the environment, revealed that different niches have evolved to be populated with different species containing unique and niche‐related genes (Figure 3E).

### The diversity of species taxonomy, body habitats, and geography shapes the various functions of Lachnospiraceae

**Figure 4:**
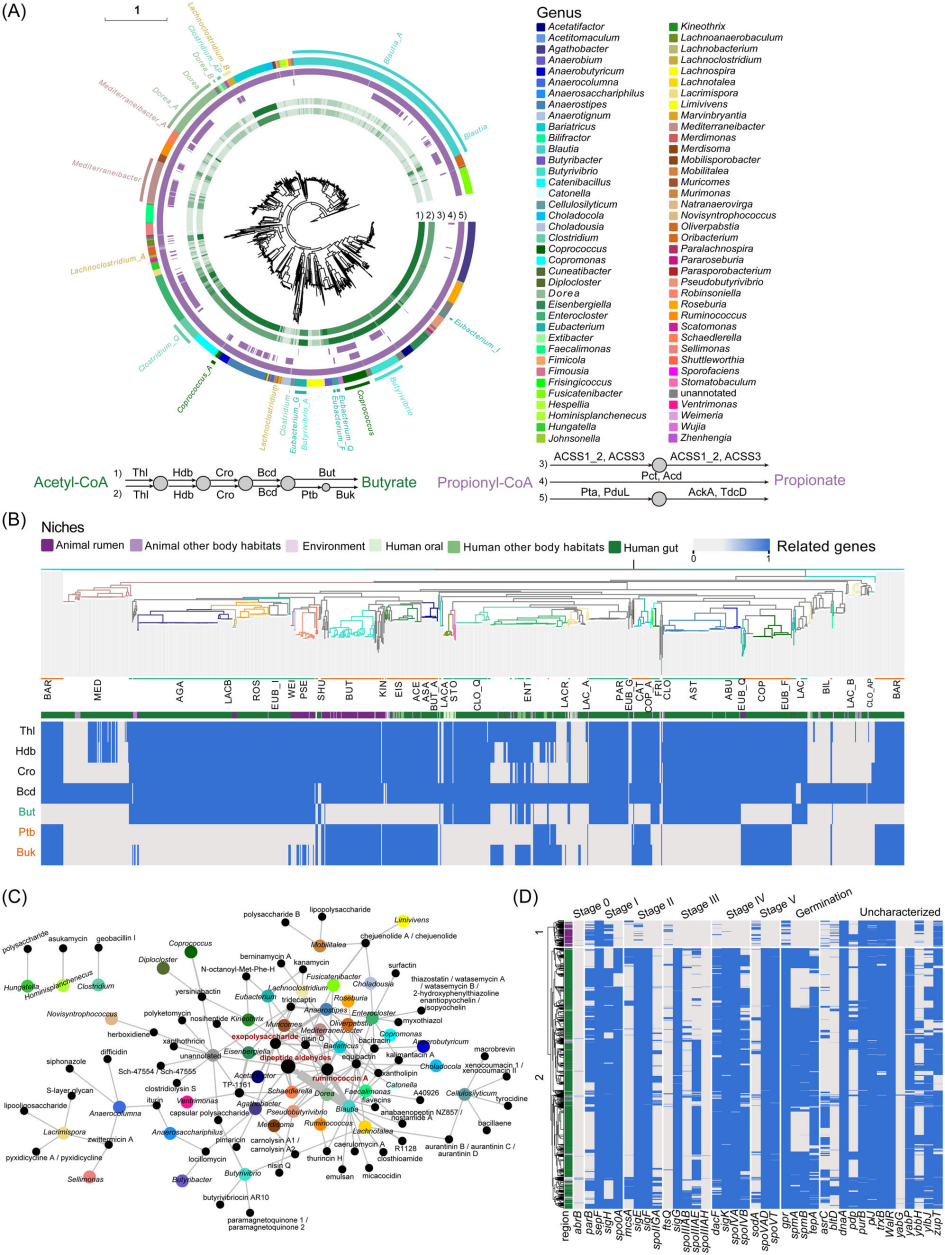
Functional profile of Lachnospiraceae. (A) Distribution of genes associated with short‐chain fatty acid production in each genome. This phylogenetic tree is consistent with Figure 1C. The two pathways for butyrate production from acetyl‐CoA (the Butyryl‐CoA transferase pathway and Butyrate kinase pathway) are presented in the first and second layers, respectively. The third to fifth layers represent the three pathways from propionyl‐CoA to propionate production. The shade of color indicates the type of genes in the pathway, that is, the integrity of the pathway. The last layer represents the genus, which is consistent with Figure 1C. Thl, thiolase; Hdb, *β*‐hydroxybutyryl‐CoA dehydrogenase; Cro, crotonase; Bcd, butyryl‐CoA dehydrogenase; But, butyryl‐CoA:acetate CoA transferase; Ptb, phosphate butyryltransferase; Buk, butyrate kinase; ACSS1_2, acetyl‐CoA synthetase; ACSS3, propionyl‐CoA synthetase; Pct, propionate CoA‐transferase; Acd, acetate‐CoA ligase (ADP‐forming); Pta, phosphate acetyltransferase; PduL, phosphate propanoyltransferase; AckA, acetate kinase; TdcD, propionate kinase. (B) Phylogenetic tree of potentially butyrate‐producing genera. The color of the clade represents the genus, and the first layer represents the niche of the genomes. The heat map is colored according to the presence or absence of genes, corresponding to the genes related to the two pathways shown on the left. ACE, *Acetatifactor*; AGA, *Agathobacter*; ABU, *Anaerobutyricum*; ASA, *Anaerosacchriphilus*; AST, *Anaerostipes*; BAR, *Bariatricus*; BAR, *Bariatricus*; BUT, *Butyrivibrio*; BUT_A, *Butyrivibrio*_A; CAT, *Catenibacillus*; CLO, *Clostridium*; CLO_AP, *Clostridium*_AP; CLO_Q, *Clostridium*_Q; COP, *Coprococcus*; COP_A, *Coprococcus*_A; EIS, *Eisenbergiella*; ENT, *Enterocloster*; EUB_F, *Eubacterium*_F; EUB_G, *Eubacterium*_G; EUB_H, *Eubacterium*_H; EUB_I, *Eubacterium*_I; EUB_Q, *Eubacterium*_Q; FRI, *Frisingicoccus*; KIN, *Kineothrix*; LACA, *Lachnoanaerobaculum*; LACB, *Lachnobacterium*; LAC, *Lachnoclostridium*; LAC_A, *Lachnoclostridium*_A; LAC_B, *Lachnoclostridium*_B; LACR, *Lacrimispora*; MED, *Mediterraneibacter*_A; PAR, *Parasporobacterium*; PSE, *Pseudobutyrivibrio*; ROS, *Roseburia*; SHU, *Shuttleworthia*; ATO, *Stomatobaculum*; WEI, *Weimeria*. (C) Network of the relationship between genera and known secondary metabolites. Genera are represented by dots of their corresponding colors, secondary metabolites are represented by black dots, and the size of the dots is related to the number. (D) Heatmap of sporulation gene distribution.

The human gut microbiota ferments carbohydrates into short‐chain fatty acids (SCFAs), especially butyrate and propionate, which are then utilized by the host. SCFAs provide energy for intestinal epithelial cells, regulate the immune system, and affect various metabolic pathways that are essential for maintaining host health. Members of the Lachnospiraceae family are considered the main producers of intestinal SCFAs. Two different pathways contribute to butyrate production from butyryl‐CoA, one dependent on butyrate kinase and one dependent on butyryl‐CoA: acetate‐CoA transferase (Table S3). The conversion of propionyl‐CoA to propionate comprises three different pathways, including one‐step reactions catalyzed by a CoA‐transferase or a CoA‐ligase and a pathway involving several intermediate steps (Table S3) [20]. We found that only 40.80% of the Lachnospiraceae genomes harbored complete butyrate pathways, whereas almost all genomes harbored complete propionate pathways that require CoA‐transferase (Figure 4A). In addition, the complete butyrate pathway predicted in the genome of Lachnospiraceae generally depends on either butyrate kinase or butyryl‐CoA: acetate‐CoA transferase, while one or more complete propionate pathways are carried on the same genome in Lachnospiraceae. *Coprococcus*, a recognized butyrate‐producing bacteria, harbors not only the complete butyrate pathway but also different propionate pathways, showing its great potential for SCFA production. We also discovered that a large number of potentially new species and new genera have the ability to produce butyrate and propionate.

To define genera that comprise members harboring the complete butyrate pathway as potential butyrate‐producing genera, we extracted the distribution of genes related to butyrate production from 26 genera (Figure 4B). We found that the integrity of the pathway was not related to the body habitat but depended on the species. Similarly, different pedigrees within the same genus exhibited differences in the completeness of pathways and pathway types, that is, there was no specificity at the genus level. For example, *Coprococcus catus* uses butyryl‐CoA: acetate CoA transferase to produce butyrate, whereas the other genomes of *Coprococcus* use the butyrate kinase pathway [8]. For *Enterocloster*, some of the genomes of *Enterocloster clostridioformis* use butyryl‐CoA: acetate CoA transferase, whereas the genome of *Enterocloster bolteae* harbors genes encoding enzymes that can produce butyrate through two pathways, but the rest of the genomes have gene deletions. This result did not match the phylogeny, indicating that genes related to butyrate production may be obtained by horizontal gene transfer.

Members of the Lachnospiraceae family have been reported in many studies to produce novel secondary metabolites [10, 21, 22]. We carried out extensive mining of secondary metabolite biosynthetic gene clusters (SMBG) using antiSMASH (V6.0.0) and explored 6688 regions (7373 SMBGs) from 1856 genomes, with a total of 32 types (Table S4). By matching these regions with the experimentally verified reference biosynthetic gene clusters (BGCs) in the MiBIG database, we identified 58 known BGCs in the Lachnospiraceae genomes (Figure 4C). The largest number of BGCs were dipeptide aldehydes, Ruminococcin A (RumA), and exopolysaccharide. Dipeptide aldehyde is a highly effective protease inhibitor that was first characterized in *Ruminococcus* sp. [23]. RumA can be used for the clinical treatment of pathogenic *Clostridium* spp. infections and has been previously characterized in *Ruminococcus gnavus* E1 [24]. We found that *Blautia* has great potential to produce dipeptide aldehyde and RumA, which not only helps them to occupy niches but also serves as an important candidate source of these biologically active products. Exopolysaccharide, mainly produced by *Anaerostipes*, can be used as cross‐feeding fermentation substrates to stimulate the growth of specific beneficial bacteria, reduce pathogen adhesion, and improve the protective effect of the intestinal barrier [25]. In addition, 90.7% of the regions exhibited no match in the MiBIG database, indicating that the structure and function have not yet been described. These results revealed that Lachnospiraceae has a great unexplored potential for the discovery of novel secondary metabolites.

### Sporulation‐mediated transmission varies among different ecological niches

Most Bacillota are known sporulating bacteria [26, 27, 28], which exhibit long‐term survival under harsh environmental conditions such as high or low temperatures, oligotrophic conditions, and exposure to drugs. In addition, spores can also promote the spread of bacteria between hosts, eventually colonizing a variety of habitats [29]. Most species of Lachnospiraceae are considered to be spore producers. Spores of Lachnospiraceae have been shown to survive ethanol treatment and germinate in the presence of bile acids [30]. Browne et al. used machine‐learning methods to identify 66 sporulation characteristic genes and further showed differential losses of these genes in different lineages of intestinal Bacillota. We found numerous deletions of characteristic genes in the genomes of animal rumen origin and the human oral habitat (Figure 4D and Figure S7). However, even though the genomes from the human gastrointestinal tract contained a relatively complete and abundant complement of these characteristic genes, some of them had lost *spo0A*, the master regulator gene essential for sporulation. This difference may indicate adaptation between different ecological niches.

### Investigating associations of the Lachnospiraceae clusters with human diseases

Metagenomics‐based approaches can identify disease‐related markers but rely mainly on database‐based annotation of reads or de novo binning, which generally lack species‐level matching, thus limiting subsequent studies. Since most biomarkers lack cultured strains, metagenomic studies most often identify disease‐related bacterial species with limited functional information and mechanistic insight. To circumvent this limitation, we used cultured genome collection to identify potential associations between health and disease.

By exploiting our genome collection for analysis of strains associated with ACVD in a Chinese cohort [7], we unveiled remarkable disparities in the abundance of bacterial strains. Specifically, we identified 56 strains from 13 clusters that were notably more prevalent in the healthy control group, while 52 strains from 14 clusters displayed a significant enrichment in the ACVD group (with an adjusted *p* value < 0.01 and |log_2_ FC| > 1, Figure S8A and Table S5). Consistent with previous studies [6, 7], we observed a higher abundance of members of the *Roseburia* genus in healthy controls, while *Ruminococcus gnavus*, considered an opportunistic pathogen, was significantly enriched in the ACVD group. Additionally, we discovered that several strains of *Lachnospira eligens*, *Acetatifactor* sp., and *Agathobacter faecis* not only played pivotal roles in coabundance networks but also exhibited negative correlations with ACVD‐enriched genomes (|*r* | > 0.3, adjusted *p* < 0.01, Figure S8B).

For intestinal inflammatory diseases, we investigated the distribution of members of the Lachnospiraceae family in the inflammatory bowel disease (IBD) cohort of the Human Microbiome Project (HMP), including Crohn's disease (CD) and ulcerative colitis (UC) patients. In CD patients, we identified 17 strains from eight clusters that were enriched, while 122 strains from 35 clusters were reduced in abundance (with an adjusted *p* value < 0.01 and |log_2_ FC| > 1, Figure S9A and Table S6). In the healthy group, we discovered an enrichment of potentially novel species such as *Acetatifactor* sp., *Choladocola* sp., *Eubacterium* sp., and strains from unidentified genera. These novel species exhibited significant negative correlations with *Enterocloster bolteae* (formerly *Clostridium bolteae*) and *Ruminococcus gnavus* (|*r* | > 0.3, adjusted *p* < 0.01, Figure S9B). In UC patients, eight strains from seven clusters were enriched, while 51 strains from 24 clusters were reduced (with an adjusted *p* value < 0.01 and |log_2_ FC| > 1, Figure S10 and Table S7). Of particular interest were two strains, GCA_009881395 and GCA_013304625, both belonging to the *Blautia wexlerae*, each displaying different patterns; GCA_009881395 was enriched in the healthy control group, whereas GCA_013304625 was enriched in the UC group (Figure S11). This observation suggests that different strains from the same species may have distinct roles in health and disease, emphasizing the importance of considering strain‐level diversity in metagenomic disease studies.

Comparing the two cohorts, we observed a marked difference in the number of strains enriched in the healthy group. While the ACVD cohort displayed a higher abundance of specific strains, the IBD cohort, encompassing CD and UC, exhibited a greater number of enriched strains (Figure S11), including *Agathobacter rectalis*, *Agathobacter* sp., *Anaerobutyricum hallii*, *Butyribacter intestini*, *Butyribacter* sp., *Eisenbergiella* sp., *Mediterraneibacter lactaris*, and *Wujia chipingensis*. These contrasting findings underscore the importance of recognizing the distinct microbial signatures associated with different diseases and reinforce the significance of conducting strain‐level analyses in metagenomic studies of diseases.

## DISCUSSION

In this study, we compiled a comprehensive genome collection of Lachnospiraceae for the analysis of taxonomic and functional diversity. Members of the Lachnospiraceae family begin to colonize early in life and increase in abundance with age [31, 32]. The genomes isolated in this study contributed with a substantial number of potentially new species, providing a preliminary indication of the unexplored taxonomic diversity of the Lachnospiriaceae family, highlighting the importance of culture‐based studies in uncovering the taxonomic richness and diversity of Lachnospiraceae. Combined with the publicly available genomes, we found that the bacteria of the Lachnospiraceae family were ubiquitous across various niches, while species‐level colonization exhibits specificity, offering valuable insights for transplantation studies using Lachnospiraceae members. The isolation of novel bacteria expanded the number of species by a factor of three compared with the presently validated 122 species, indicating that the species diversity of Lachnospiraceae so far has been underestimated. Notably, eight genera of Lachnospiraceae exhibited greater diversity, showing distinct branches in the phylogenetic tree, along with variations in short‐chain fatty acid (SCFA) synthesis pathways and disease associations. We propose that taxonomic studies on potentially new species are warranted to provide valid names, and reclassification of multi‐lineage genera is necessary to refine the taxonomy.

Regarding functional exploration, we constructed a 1.5 M gene and a 1.4 M protein catalog, with potentially novel species playing a major role, shedding new light on Lachnospiraceae metabolism and biology. Human gut‐derived Lachnospiraceae strains were found to encompass nearly half of the functions found in the human gut microbiota, further emphasizing their essential roles within this ecosystem. We performed pan‐genome analyses separately for genera and species, enriching our understanding of well‐studied [33, 34] and poorly studied members.

The complete biosynthesis pathway of SCFA was constructed for all genomes, and a large number of SMBGs were predicted, emphasizing the potential ecological importance of Lachnospiraceae in the human gut. Studies have shown that species such as *Blautia* and *Roseburia*, which are usually considered beneficial species, are major SCFA producers [35]. In this study, we observed that nearly all Lachnospiraceae members possess the capacity to produce propionate, while most strains were butyrate producers with niche‐dependent specificity.

Although the isolation of microbial secondary metabolites has mainly focused on specific organisms present in the environment, especially *Streptomyces*, *Aspergillus*, and *Pseudomonas*, host‐associated microbes may also contribute to the production of interesting secondary metabolites [36, 37, 38]. In the MiBIG database, 10 of 1926 experimentally validated secondary metabolites were first discovered and extracted from four genera of Lachnospiraceae. Our study predicted that 7373 gene clusters from 1856 genomes have the potential to produce interesting secondary metabolites, indicating that Lachnospiraceae has a surprising ability to produce such compounds, most of which remain to be fully characterized. While we successfully predicted a vast number of gene clusters with potential for secondary metabolite production, we did not specifically address the presence of individual metabolites like RumA or exopolysaccharides in the bacterial cultures. Our future studies can indeed build upon the findings and focus on isolating specific strains within the Lachnospiraceae family to characterize their secondary metabolites more comprehensively. Such efforts might involve culture‐based methods to confirm the presence of specific metabolites and investigate their biological roles.

Spores are stress‐resistant structures formed by Bacillota [27]. Previous research on microbial spores has not been limited to pathogens [39, 40] but has also focused on probiotic bacteria [41, 42]. With the development of human intestinal microbial culturomics in recent years, an increasing number of strains and high‐quality genomes have been established, enabling studies of spore production by human intestinal microbes [26, 28, 30]. Several studies have suggested using spore preparations as an alternative to traditional fecal microbiota transplantation (FMT) for the treatment of *Clostridioides difficile* infection and IBD [43, 44, 45]. The purification process of spores selectively eliminates bacteria, fungi, and viruses by mixing with ethanol, which is safer than the traditional FMT. In addition, spore preparations can remain active for germination and replication in the recipients. Through gene prediction, we found that most members of the human gut Lachnospiraceae are able to form spores. However, additional experiments are needed to validate the actual spore germination potential and safety of Lachnospiraceae spores for therapeutic interventions. Our study also yielded intriguing insights into the absence of sporulation‐related genes within Lachnospiraceae species inhabiting the animal rumens and the human oral cavity. The observation of this absence is indicative of the unique ecological niches occupied by these bacteria, and it likely reflects the selective pressures and specific requirements they face in their respective environments. Furthermore, sporulation is closely linked to the dispersal strategies of microorganisms. While our findings suggest a lack of sporulation‐related genes, we acknowledge that additional research is essential to provide comprehensive validation and a deeper understanding of its implications.

Our study demonstrated that the cultured genome collection of Lachnospiraceae enhances the resolution of disease‐related genomes and provides a basis for selecting potentially effective strains for intervention. Lachnospiraceae, a prominent family of bacteria within the human gut microbiome, has gained increasing attention due to its role in maintaining gut homeostasis and its impact on host health. We found that most of the strains enriched in the control groups were of unknown species, further emphasizing the importance of new species. Furthermore, compared with the use of existing databases, the use of cultured genomes as a reference allows the identification of markers for specific known strains, enabling subsequent functional validation. The correlation observed between Lachnospiraceae abundance and the prevalence of ACVD and IBD suggests that these bacteria may exert a protective influence in relation to these diseases. While further research is needed to establish causation and elucidate the underlying mechanisms, our findings offer promising insights.

## CONCLUSION

Our comprehensive genomic and functional analyses of Lachnospiraceae strains enable targeted isolation and functional screening, providing a promising avenue for the development of novel probiotics and antibiotic alternatives. This research significantly contributes to our understanding of the unexplored potential of Lachnospiraceae in improving human health and offers valuable insights for future probiotic‐related investigations.

## METHODS

### Evaluation of novelty of CGR2 genomes

We downloaded the 16S rRNA gene sequences of all 122 type strains included in the LPSN with valid and correct name at the beginning of this study, that is, July 2021. The 16S rRNA gene sequences of 756 CGR2 genomes were extracted using Barrnap (version 0.9). Pairwise BLASTn was performed using BLAST 2.12.0+ with an identity of 98.7% as a species‐level cut‐off and 94.5% as a genera cut‐off [18]. The 16S rRNA gene sequences of potentially new genera in CGR2 were clustered by usearch (v11.0.667) [46] to obtain the OTU at the species and genus levels (using options: ‐‐id 0.987 and 0.945, respectively). Genus‐level representative 16S rRNA gene sequences were extracted and aligned using MAFFT v7.310 [47] and trimmed using trimAl v1.4. rev 22 [48] with the auto option. The phylogenetic tree was reconstructed using the maximum‐likelihood method with FastTree Version 2.1.3 SSE3 [49].

### Genome collection and quality assessment

To establish a collection of isolated genomes of Lachnospiraceae, we downloaded all isolated genomes labeled as Lachnospiraceae from the NCBI [50] and IMG [51] databases (July 2021). We performed this collection with currently established cultured‐based genomes of CGR2 and UHGG [52]. Traceability investigations were conducted for all genomes, including host and country information (animal habitats include cow, sheep, mouse, pig, chicken, dog, llama, wallaby, and wood turtle). To avoid genome duplication caused by synchronization of the database, we used fastANI (v1.32) [53] to compare the genomes for different data sets, and only one of the genomes was retained when the genomes were highly similar (pairwise ANI was 100%) and shared common strain names. Only genomes with >90% completeness and <5% contamination, as estimated by CheckM (v1.1.2, “lineage_wf” workflow) [54] were defined as high‐quality genomes and were retained for further analysis.

### Phylogenetic and taxonomic analyses

Genomes that shared ≥95% ANI were considered the same species [55]. Thus, we employ fastANI (v1.32) [53] to calculate pairwise ANI values between genomes and generate a matrix (‐‐matrix). Subsequently, we conducted a hierarchical clustering analysis using the “hclust” function from the R package (method = “complete”). The resulting hierarchical clustering dendrogram was then divided into clusters based on the 95% ANI threshold using the “cutree” function (*h* = 0.05). dDDH value was calculated by GGDC (https://ggdc.dsmz.de/ggdc.php#) [56].

Taxonomic annotation of each genome was performed with GTDB‐Tk [57] (v2.3.2, database release214 [58]) using the “classify_wf” function and default parameters. Any lineage without a valid name was considered to represent a potentially new species or genus. The additional letter suffix of the genus name indicates high phylogenetic diversity.

PhyloPhlAn 3.0 [59] was used to perform a phylogenetic analysis of 1868 genomes. The process involved several specific steps. Initially, DIAMOND [60] was utilized to identify marker genes. This was achieved by mapping the amino acid sequences from the 1868 genomes with the PhyloPhlAn 3.0 database, which includes a set of 400 universally marker genes present in all bacteria and archaea. Subsequently, the mapping results were processed through MAFFT for alignment optimization. Alignments were further refined using trimAl. Finally, a maximum likelihood tree was constructed through the application of IQ‐TREE [61], followed by a refinement step using RAxML [62]. All phylogenetic trees in this study were visualized and annotated with the online tool EVOLVIEW v2 [63].

### Construction of the nonredundant gene/protein catalog and pan‐genome analyses

In the initial phase of our analysis, the 1868 genomes were annotated by Prokka v1.14.6 [64] with default settings to predict both nucleotide and protein sequences associated with the genes present in these genomes.

Nucleotide sequences were used for CD‐HIT v4.6.3 [65] to generate gene catalogs [65], utilizing specific parameters such as ‐c 0.95 and ‐aS 0.9, which defined stringent criteria of 95% protein identity and 90% coverage. This step facilitated the generation of a nonredundant gene catalog, ensuring that each gene was uniquely represented and eliminating redundancy in the data set.

In parallel, we also turned to the construction of the protein catalog. To achieve this, protein sequences were used for “linclust” function of MMseqs. 2 (Version 13.45111) [66], employing a set of defined parameters, including “‐‐cov‐mode 1 ‐c 0.8 ‐‐kmer‐per‐seq. 80 ‐‐min‐seq‐id 0.95,” which defined previously used criteria of 95% protein identity and 80% coverage [52].

Pan‐genome analyses were carried out by Roary v3.7.0, with option “‐i 90” [19] to identify the core and cloud genes of family, genera, and species. In terms of eight multilineage genera, the gene‐genome matrix generated by Roary that included the presence/absence profile of each gene family for all genomes was extracted, and the R function “vegdist” was used to calculate the pairwise Jaccard index between genomes. Heatmap visualization was performed by the ComplexHeatmap R package [67].

Parsnp v1.5.0 [68] was employed with default settings to collect SNPs from all genomes of *Agathobacter rectalis* and generate an SNP‐based phylogenetic tree. Variants were annotated using SnpEff v5.1 [69], which reports their predicted impact on the protein (HIGH, MODERATE, LOW, or MODIFIER).

### Functional characterization

The function profile of the nonredundant gene catalog was carried out by eggNOG‐mapper v2 [70] (eggNOG database version: 5.0.2 [71]). KEGG ORTHOLOGY (KO) was extracted from the eggNOG‐mapper results and visualized in iPath3 [72]. To identify KOs that differed significantly between known and novel clusters, linear discriminant analysis effect sizes (LEfSes) were determined using the Huttenhower Lab Galaxy module. For the analysis process of LEfSe, the sum of gene values per genome was normalized to 1 M. The input parameters were as follows: alpha value for the pairwise Wilcoxon test between subclasses was 0.01, logarithmic linear discriminant analysis score threshold for discriminative features was 2.0.

The acetyl‐CoA‐to‐butyrate and propionyl‐CoA‐to propionate biosynthesis pathways were generated according to previous studies [20, 73], and the protein sequences of associated enzymes were extracted from the KEGG database to construct a small database. The enzyme Commission numbers of relevant enzymes are shown in Table S3. To better describe the potential ability of the strains to participate in the production of butyrate and propionate, we BLASTed the gene sequences of each strain on the database constructed above (blastp, cut‐off 1e‐2, identity ≥ 60%, coverage ≥ 50%). If a genome could be annotated with enzymes for all steps in the pathway in Figure 3A, it was defined as having a complete pathway.

A total of 7373 SMBGs were mined by antiSMASH 6.0 [74], a tool that can detect BGCs and characterize known functions. The relationship between the known function SMBGs and their regional genome was displayed using Cytoscape (v3.8.2) [75].

A previous study has proposed 66 sporulation characteristic genes [28]. We searched for these gene names in the prokka annotation results to obtain the distribution of the characteristic genes in each genome.

### Identification of disease‐associated markers of Lachnospiraceae

The clean data of 385 metagenomes (171 from healthy control individuals and 214 from individuals with ACVD) were downloaded from the European Bioinformatics Institute (EBI) database with the accession number ERP023788 of one study of human gut microbiome association with ACVD [7]. Human gut metagenome sequencing data of the IBDMDB study [76] were downloaded (https://portal.hmpdacc.org/), and the metadata can be found through https://ibdmdb.org/results/HMP2/.

To calculate the abundance of Lachnospiraceae genomes across the samples, we built a Kraken2/Bracken database (options: ‐k 31 ‐l 100) with 1868 genomes of the Lachnospiraceae family. For each sample, reads assignment was performed using Kraken2 v2.1.2 and Bracken v 2.6.1. A threshold of 0.001% relative abundance and at least 10% occurrence was assigned to define the presence of the genome in the sample.

Abundances were calculated, and no reads mapping samples or genomes were filtered out using R software. EdgeR, a negative binomial‐based R package, was used to identify genomes with significantly different abundances and select genomes with adjusted *p* value < 0.01 and |log_2_ FC| > 1. R function “corr. test” to conduct bacteria co‐occurrence analysis.

## AUTHOR CONTRIBUTIONS

Yuanqiang Zou and Liang Xiao conceived the study. Xiaoqian Lin, Zhinan Wu, Lingne Li, Yuhao Wang, and Dingyang Wen collected the genomes. Xiaoqian Lin, Tongyuan Hu, Xudong Liu, Wenxi Li, and Hewei Liang analyzed the data. Yuanqiang Zou, Xin Jin, Xun Xu, Huanming Yang, and Jian Wang contributed analysis tools. Xiaoqian Lin, Yuanqiang Zou, Liang Xiao, and Karsten Kristiansen wrote the paper. Karsten Kristiansen revised the paper. All authors have read the final manuscript and approved it for publication.

## CONFLICT OF INTEREST STATEMENT

The authors declare no conflict of interest.

## ETHICS STATEMENT

No animals or humans were involved in this study.

## Supporting information


**Figure S1.** Assessment of the digital DNA‐DNA hybridization values for the clusters at the species level.
**Figure S2.** Genomic and 16S rRNA similarity with the type strains.
**Figure S3.** Proportion of genomes isolated from the three ecological niches.
**Figure S4.** Investigating functional profiles by constructing a protein catalog.
**Figure S5.** Pan‐genome analyses of specific genera.
**Figure S6.** SNP analysis of *Agathobacter rectalis*.
**Figure S7.** Principal Coordinates Analysis of genomes from different niches according to the presence or absence of sporulation characteristic genes.
**Figure S8.** Co‐occurrence network deduced from 108 Lachnospiraceae genomes enriched in ACVD and control cohorts.
**Figure S9.** Co‐occurrence network deduced from 139 Lachnospiraceae genomes enriched in CD and control cohorts.
**Figure S10.** Co‐occurrence network deduced from 59 Lachnospiraceae genomes enriched in UC and control cohorts.
**Figure S11.** Enrichment of 58 clusters in ACVD or IBD cohort patients and healthy individuals.

**Table S1.** The genome and taxonomic annotation information of the 1868 strains.
**Table S2.** Number of genomes in 387 clusters.
**Table S3.** The enzyme commission numbers of key enzymes in the SCFA synthesis pathway.
**Table S4.** The 7373 explored SMBGs.
**Table S5.** Differentially enriched genomes of the ACVD cohorts.
**Table S6.** Differentially enriched genomes of the CD cohorts.
**Table S7.** Differentially enriched genomes of the UC cohorts.

## Data Availability

All the genomes used in this study are available in public repositories, and the accession numbers or references are provided in Table S1. For all public metagenomic data used in this study, the web links or references are provided in the Materials and Methods Section. The data and scripts used are saved in GitHub https://github.com/Linxiaoqianv/Lachnospiraceae_iMeta. Supplementary materials (figures, tables, graphical abstract, and source data) may be found in the online DOI or iMeta Science http://www.imeta.science/.
