# Supplementary material for: Isolation of potentially novel species expands the genomic and functional diversity of Lachnospiraceae
Source: Imeta. 2024 Feb 13;3(2):e174. doi: 10.1002/imt2.174 (PMC11170972; doi:10.1002/imt2.174)
Supplement: Supplementary file 1 — Figure S1. Assessment of the digital DNA‐DNA hybridization values for the clusters at the species level. Figure S2. Genomic and 16S rRNA similarity with the type strains. Figure S3. Proportion of genomes isolated from the three ecological niches. Figure S4. Investigating functional profiles by constructing a protein catalog. Figure S5. Pan‐genome analyses of specific genera. Figure S6. SNP analysis of Agathobacter rectalis. Figure S7. Principal Coordinates Analysis of genomes from different niches according to the presence or absence of sporulation characteristic genes. Figure S8. Co‐occurrence network deduced from 108 Lachnospiraceae genomes enriched in ACVD and control cohorts. Figure S9. Co‐occurrence network deduced from 139 Lachnospiraceae genomes enriched in CD and control cohorts. Figure S10. Co‐occurrence network deduced from 59 Lachnospiraceae genomes enriched in UC and control cohorts. Figure S11. Enrichment of 58 clusters in ACVD or IBD cohort patients and healthy individuals. [file IMT2-3-e174-s002.docx]

**Supporting information to:**

**Isolation of potentially novel species expands the genomic and functional diversity of Lachnospiraceae**

**Running title**: The genomic and functional diversity of Lachnospiraceae

Xiaoqian Lin^1, 2^, Tongyuan Hu^1^, Zhinan Wu^1, 3^, Lingne Li^1^, Yuhao Wang^1^, Dingyang Wen^1^, Xudong Liu^1, 3^, Wenxi Li^1, 2^, Hewei Liang^1^, Xin Jin^1^, Xun Xu^1^, Jian Wang^1, 4^, Huanming Yang^1, 4^, Karsten Kristiansen^1, 5, 6*^, Liang Xiao^1, 3, 5, 7*^, and Yuanqiang Zou^1, 5, 6, 7*^

^1^ BGI Research, Shenzhen 518083, China

^2^ School of Bioscience and Biotechnology, South China University of Technology, Guangzhou 510006, China

^3^ College of Life Sciences, University of Chinese Academy of Sciences, Beijing 100049，China

^4^ James D. Watson Institute of Genome Sciences, Hangzhou 310058, China

^5^ Lars Bolund Institute of Regenerative Medicine Qingdao-Europe Advanced Institute for LifeSciences, BGI Research, Qingdao 266555, China

^6^ Laboratory of Genomics and Molecular Biomedicine, Department of Biology, University of Copenhagen, Universitetsparken 13, 2100 Copenhagen, Denmark

^7^ Shenzhen Engineering Laboratory of Detection and Intervention of human intestinal microbiome, BGI-Shenzhen, Shenzhen 518083, China

*Correspondence:

kk@bio.ku.dk (Karsten Kristiansen), xiaoliang@genomics.cn (Liang Xiao), and zouyuanqiang@genomics.cn (Yuanqiang Zou)

**Supplementary Figures**

Figure S1. Assessment of the digital DNA-DNA hybridization values for the clusters at the species level.

Figure S2. Genomic and 16S rRNA similarity with the type strains. A dot represents a cluster. The genomes are divided into 5 parts according to the threshold, and the cluster is colored when all the genomes in the cluster have a consistent distribution part.

Figure S3. Proportion of genomes isolated from the three ecological niches. (A-B) A dot represents a genus (A) or cluster (B), and the distance from the three vertices represents the proportion of genomes (within that genus or cluster) isolated from the three niches. For example, the vertices indicate that all genomes are isolated from that niche, and the center of the triangle represents an equal number of genomes isolated from the three niches.

Figure S4. Investigate functional profiles by constructing protein catalog. (A) The 1.4M protein catalog of 1,868 genomes. (B) The significant differences of KOs between potentially novel and known species. (C) Combined global map of metabolic pathways (map01100). Compounds are represented by dots. The pathways colored orange, green, or blue indicate human gut Lachnospiraceae only, gut microbiota only, and both, respectively. The pathways which are more complete are highlighted with text. (D) The Lachnospiraceae genomes isolated from the human gut perform approximately half of the functions of the human gut microbiota. The numbers represent the number of KO.

Figure S5. Pan-genome analyses of specific genera. (A) Statistics on core genomes and pan-genomes of genera with a total number of genomes greater than 10. (B) 6 genera (including multi-lineages) containing more than 10 potentially new species genomes. (C) Pan-genome accumulation curves of 6 genera before (dotted line) and after (solid line) inclusion of potentially new species.

Figure S6. SNP analysis of *Agathobacter rectalis*. (A) The top ten gene presentations were selected based on the number of variants that had a high and moderate effect on the protein. (B) The variant ratio at different genome positions of the 4 clades. The genomic position is colored according to the mutation type.

Figure S7. Principal Coordinates Analysis of genomes from different niches according to the presence or absence of sporulation characteristic genes. The solid line range represents the 95% confidence interval.


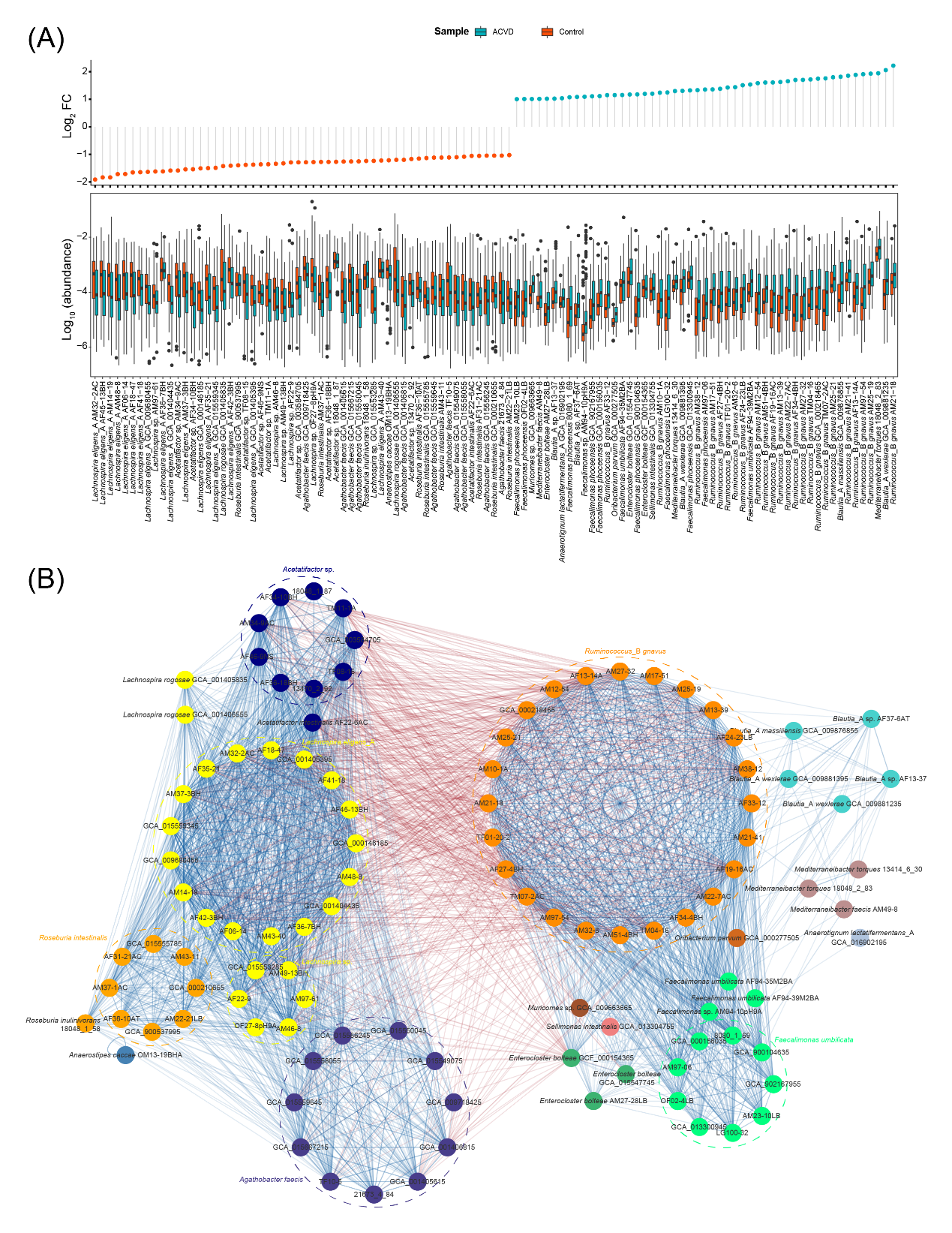


Figure S8. A Co-occurrence network deduced from 108 Lachnospiraceae genomes enriched in ACVD and control cohorts. (A) 108 genomes with significantly different abundances in health and ACVD. The left panel shows the abundance of the genomes in healthy individuals and the ACVD group, respectively, and the right panel shows the log_2_ FC. (B) Nodes depict genomes and their taxonomy. Lines represent |r| value > 0.3 and are colored according to correlation.


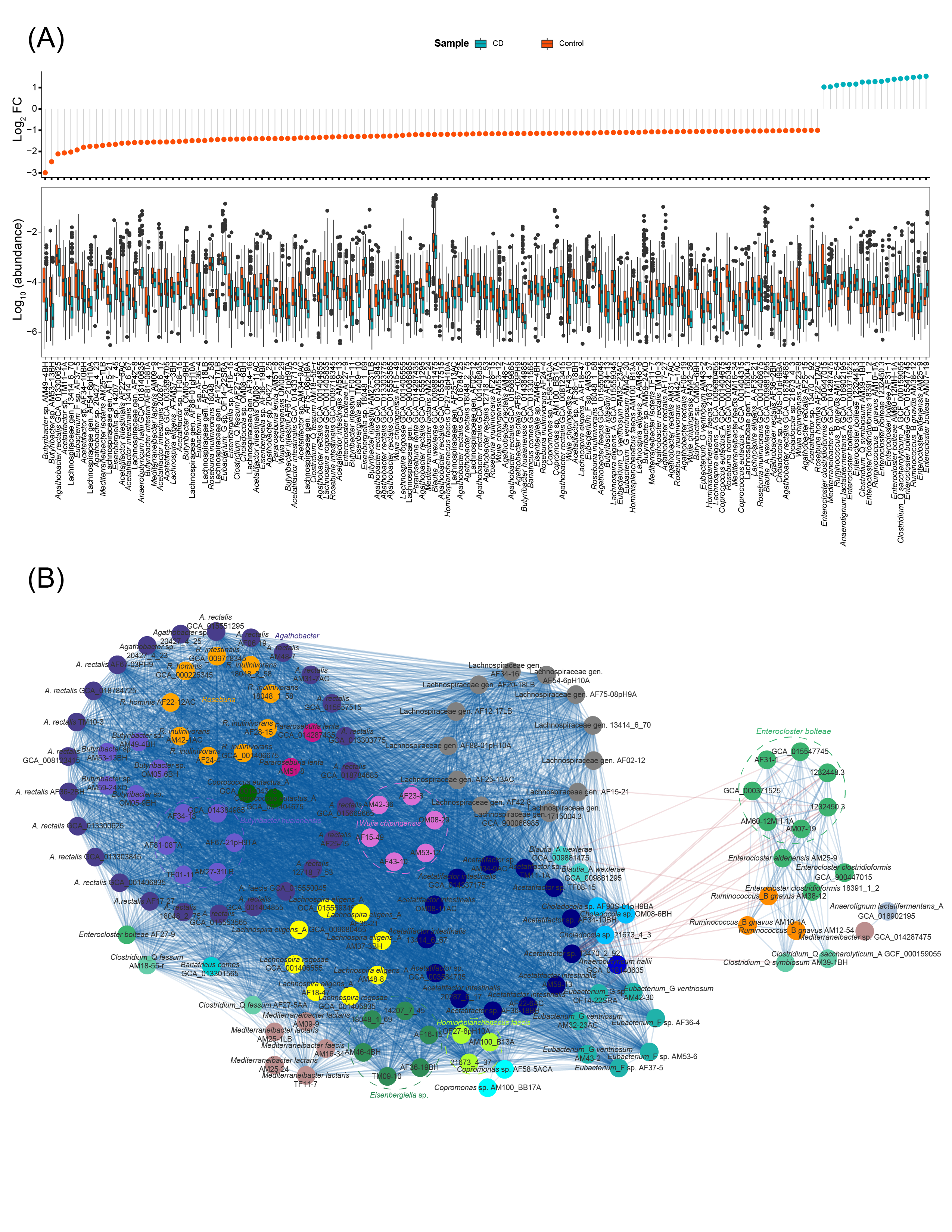


Figure S9. A Co-occurrence network deduced from 139 Lachnospiraceae genomes enriched in CD and control cohorts. (A) 139 genomes with significantly different abundances in health and CD. The left panel shows the abundance of the genomes in healthy individuals and the CD group, respectively, and the right panel shows the log_2_ FC. (B) Nodes depict genomes and their taxonomy. Lines represent |r| value > 0.3 and are colored according to correlation.


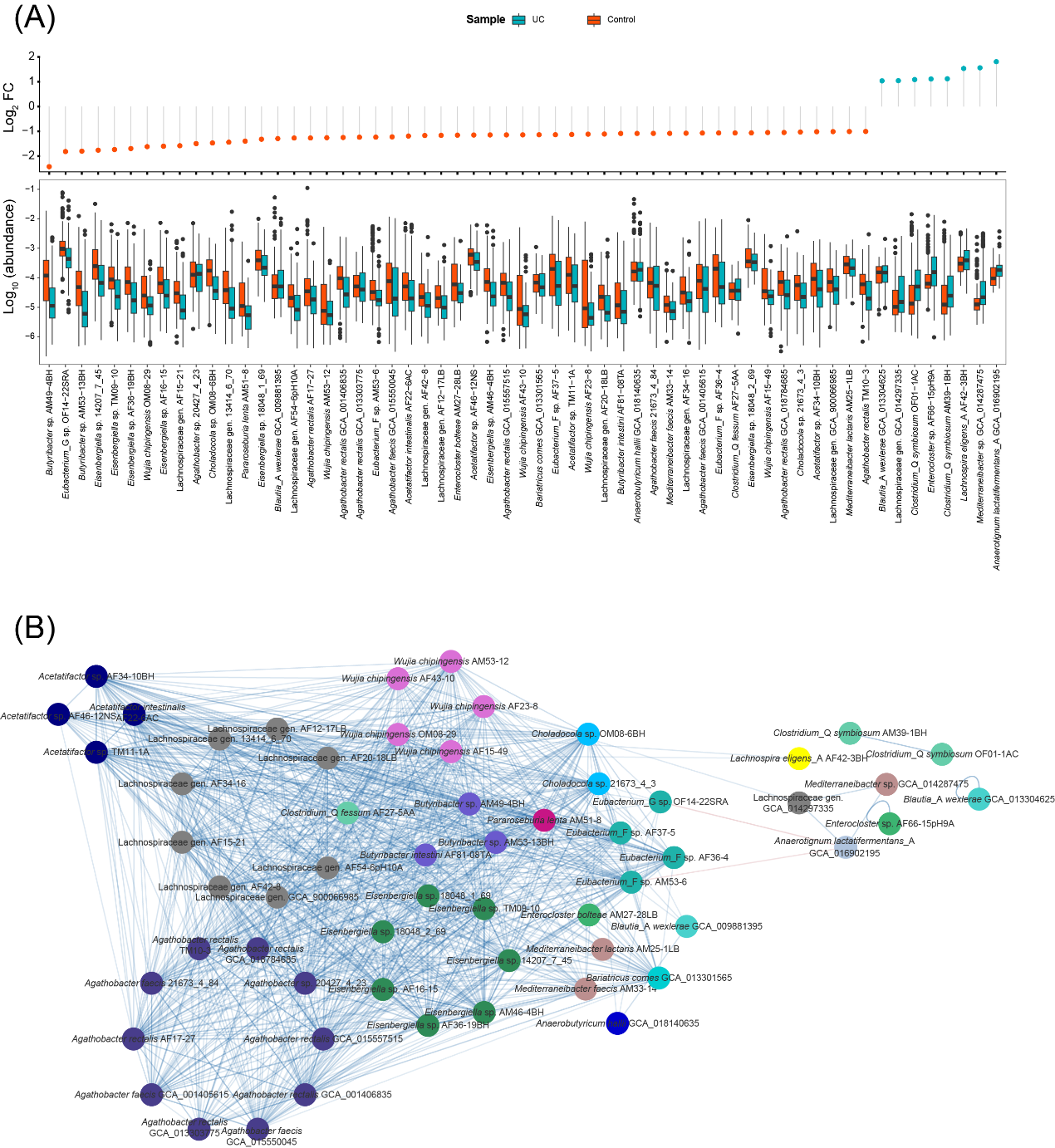


Figure S10. A Co-occurrence network deduced from 59 Lachnospiraceae genomes enriched in UC and control cohorts. (A) 59 genomes with significantly different abundances in health and UC. The left panel shows the abundance of the genomes in healthy individuals and the UC group, respectively, and the right panel shows the log_2_ FC. (B) Nodes depict genomes and their taxonomy. Lines represent |r| value > 0.3 and are colored according to correlation.

Figure S11. Enrichment of 58 clusters in ACVD or IBD cohort patients and healthy individuals. log_2_ FC > 1 means enrichment in patients, log_2_ FC < 1 means enrichment in healthy individuals. The dots within the figure represent individual genomes within each cluster, and their color corresponds to the taxonomic novelty of the genome.

**Supplementary references (for Table S1)**

1. Lagkouvardos, Ilias, Rüdiger Pukall, Birte Abt, Bärbel U. Foesel, Jan P. Meier-Kolthoff, Neeraj Kumar, Anne Bresciani, et al. 2016. “The Mouse Intestinal Bacterial Collection (miBC) provides host-specific insight into cultured diversity and functional potential of the gut microbiota.” *Nature Microbiology* 1: 1−15. <https://doi.org/10.1038/nmicrobiol.2016.131>

2. Forster, Samuel C., Nitin Kumar, Blessing O. Anonye, Alexandre Almeida, Elisa Viciani, Mark D. Stares, Matthew Dunn, et al. 2019. “A human gut bacterial genome and culture collection for improved metagenomic analyses.” *Nature Biotechnology* 37: 186−92. <https://doi.org/10.1038/s41587-018-0009-7>

3. Greening, R. C., J. A. Z. Leedle. 1989. “Enrichment and isolation of *Acetitomaculum ruminis*, gen. nov., sp. nov.: acetogenic bacteria from the bovine rumen.” *Archives of Microbiology* 151: 399−406. <https://doi.org/10.1007/BF00416597>

4. Rosero, Jaime A, Jirˇí Killer, Hana Sechovcová, Jakub Mrázek, Oldrˇich Benada, Katerˇina Fliegerová, Jaroslav Havlík, Jan Kopečný. 2016. “Reclassification of *Eubacterium rectale* (Hauduroy et al. 1937) Prevot 1938 in a new genus *Agathobacter* gen. nov. as *Agathobacter rectalis* comb. nov., and description of *Agathobacter ruminis* sp. nov., isolated from the rumen contents of sheep and cows.” *International Journal of Systematic and Evolutionary Microbiology* 66: 768−73. <https://doi.org/10.1099/ijsem.0.000788>

5. Aggarwala, Varun, Ilaria Mogno, Zhihua Li, Chao Yang, Graham J. Britton, Alice Chen-Liaw, Josephine Mitcham, et al. 2021. “Precise quantification of bacterial strains after fecal microbiota transplantation delineates long-term engraftment and explains outcomes.” *Nature Microbiology* 6: 1309−18. <https://doi.org/10.1038/s41564-021-00966-0>

6. Yang, Chao, Ilaria Mogno, Eduardo J. Contijoch, Joshua N. Borgerding, Varun Aggarwala, Zhihua Li, Sophia Siu, et al. 2020. “Fecal IgA levels are determined by strain-level differences in *Bacteroides ovatus* and are modifiable by gut microbiota manipulation.” *Cell Host & Microbe* 27: 467−75. e6. <https://doi.org/10.1016/j.chom.2020.01.016>

7. Poyet, M., M. Groussin, S. M. Gibbons, J. Avila-Pacheco, X. Jiang, S. M. Kearney, A. R. Perrotta, et al. 2019. “A library of human gut bacterial isolates paired with longitudinal multiomics data enables mechanistic microbiome research.” *Nature Medicine* 25: 1442−52. <https://doi.org/10.1038/s41591-019-0559-3>

8. Sorbara, Matthew T., Eric R. Littmann, Emily Fontana, Thomas U. Moody, Claire E. Kohout, Mergim Gjonbalaj, Vincent Eaton, Ruth Seok, Ingrid M. Leiner, Eric G. Pamer. 2020. “Functional and genomic variation between human-derived isolates of Lachnospiraceae reveals inter- and intra-species diversity.” *Cell Host & Microbe* 28: 134−46. e4. <https://doi.org/10.1016/j.chom.2020.05.005>

9. Browne, Hilary P., Samuel C. Forster, Blessing O. Anonye, Nitin Kumar, B. Anne Neville, Mark D. Stares, David Goulding, Trevor D. Lawley. 2016. “Culturing of 'unculturable' human microbiota reveals novel taxa and extensive sporulation.” *Nature* 533: 543−6. <https://doi.org/10.1038/nature17645>

10. Patil, Yogita, Madan Junghare, Michael Pester, Nicolai Muller, Bernhard Schink. 2015. “*Anaerobium acetethylicum* gen. nov., sp. nov., a strictly anaerobic, gluconate-fermenting bacterium isolated from a methanogenic bioreactor.” *International Journal of Systematic and Evolutionary Microbiology* 65: 3289−96. <https://doi.org/10.1099/ijsem.0.000410>

11. Perraudeau, Fanny, Paul McMurdie, James Bullard, Andrew Cheng, Colleen Cutcliffe, Achal Deo, John Eid, et al. 2020. “Improvements to postprandial glucose control in subjects with type 2 diabetes: a multicenter, double blind, randomized placebo-controlled trial of a novel probiotic formulation.” *BMJ Open Diabetes Research & Care* 8: e001319. <https://doi.org/10.1136/bmjdrc-2020-001319>

12. Shetty, Sudarshan A., Simone Zuffa, Thi Phuong Nam Bui, Steven Aalvink, Hauke Smidt, Willem M. De Vos. 2018. “Reclassification of *Eubacterium hallii* as *Anaerobutyricum hallii* gen. nov., comb. nov., and description of *Anaerobutyricum soehngenii* sp. nov., a butyrate and propionate-producing bacterium from infant faeces.” *International Journal of Systematic and Evolutionary Microbiology* 68: 3741−6. <https://doi.org/10.1099/ijsem.0.003041>

13. Holdeman, Lillian V., W. E. C. Moore. 1974. “New genus, *Coprococcus*, twelve new species, and emended descriptions of four previously described species of bacteria from human feces.” *International Journal of Systematic Bacteriology* 24: 260−77. <https://doi.org/10.1099/00207713-24-2-260>

14. Tanno, Hiroki, Tadashi Fujii, Katsuaki Hirano, Shintaro Maeno, Takashi Tonozuka, Mitsuo Sakamoto, Moriya Ohkuma, Takumi Tochio, Akihito Endo. 2021. “Characterization of fructooligosaccharide metabolism and fructooligosaccharide-degrading enzymes in human commensal butyrate producers.” *Gut Microbes* 13: 1−20. <https://doi.org/10.1080/19490976.2020.1869503>

15. Shetty, Sudarshan A., Jarmo Ritari, Lars Paulin, Hauke Smidt, Willem M. De Vos. 2017. “Complete genome sequence of *Eubacterium hallii* strain L2-7.” *Genome Announcements* 5: e01167-17. <https://doi.org/10.1128/genomeA.01167-17>

16. Barcenilla, Adela, Susan E. Pryde, Jennifer C. Martin, Sylvia H. Duncan, Colin S. Stewart, Colin Henderson, Harry J. Flint. 2000. “Phylogenetic relationships of butyrate-producing bacteria from the human gut.” *Applied and Environmental Microbiology* 66: 1654−61. <https://doi.org/10.1128/AEM.66.4.1654-1661.2000>

17. Wylensek, David, Thomas C. A. Hitch, Thomas Riedel, Afrizal Afrizal, Neeraj Kumar, Esther Wortmann, Tianzhe Liu, et al. 2020. “A collection of bacterial isolates from the pig intestine reveals functional and taxonomic diversity.” *Nature Communications* 11: 6389. <https://doi.org/10.1038/s41467-020-19929-w>

18. Hardman, John K., Thressa C. Stadtman. 1960. “Metabolism of omega-acids. II. Fermentation of delta-aminovaleric acid by *Clostridium aminovalericum* n. sp.” *Journal of Bacteriology* 79: 549−52. <https://doi.org/10.1128/jb.79.4.549-552.1960>

19. Doyle, Conor J., David Gleeson, Paul W. O'Toole, Paul D. Cotter. 2017. “Impacts of seasonal housing and teat preparation on raw milk microbiota: a high-throughput sequencing study.” *Applied and Environmental Microbiology* 83: e02694-16. <https://doi.org/10.1128/AEM.02694-16>

20. Ueki, Atsuko, Yoshimi Ohtaki, Nobuo Kaku, Katsuji Ueki. 2016. “Descriptions of *Anaerotaenia torta* gen. nov., sp. nov. and *Anaerocolumna cellulosilytica* gen. nov., sp. nov. isolated from a methanogenic reactor of cattle waste and reclassification of *Clostridium aminovalericum*, *Clostridium jejuense* and *Clostridium xylanovorans* as *Anaerocolumna* species.” *International Journal of Systematic and Evolutionary Microbiology* 66: 2936−43. <https://doi.org/10.1099/ijsem.0.001123>

21. Goeker, Markus. The One Thousand Microbial Genomes Phase 4 Project (KMG-4) sequencing the most valuable type-strain genomes for metagenomic binning, comparative biology and taxonomic classification. <https://doi.org/10.25585/1488430>.

22. Mechichi, T., M. Labat, J. -L. Garcia, P. Thomas, B. K. C. Patel. 1999. “Characterization of a new xylanolytic bacterium, *Clostridium xylanovorans* sp. nov.” *Systematic and Applied Microbiology* 22: 366−71. <https://doi.org/10.1016/S0723-2020(99)80044-7>

23. Kim, Wonduck, Sung-Hyun Yang, Mi-Jeong Park, Jihye Oh, Jung-Hyun Lee, Kae Kyoung Kwon. 2019. “*Anaerosacchariphilus polymeriproducens* gen. nov., sp. nov., an anaerobic bacterium isolated from a salt field.” *International Journal of Systematic and Evolutionary Microbiology* 69: 1934−40. <https://doi.org/10.1099/ijsem.0.003404>

24. Liu, Chang, Meng-Xuan Du, Rexiding Abuduaini, Hai-Ying Yu, Dan-Hua Li, Yu-Jing Wang, Nan Zhou, et al. 2021. “Enlightening the taxonomy darkness of human gut microbiomes with a cultured biobank.” *Microbiome* 9: 119. <https://doi.org/10.1186/s40168-021-01064-3>

25. Schwiertz, Andreas, Georgina L. Hold, Sylvia H. Duncan, Barbel Gruhl, Matthew D. Collins, Paul A. Lawson, Harry J. Flint, Michael Blaut. 2002. “*Anaerostipes caccae* gen. nov., sp. nov., a new saccharolytic, acetate-utilising, butyrate-producing bacterium from human faeces.” *Systematic and Applied Microbiology* 25: 46−51. <https://doi.org/10.1078/0723-2020-00096>

26. Tanoue, Takeshi, Satoru Morita, Damian R. Plichta, Ashwin N. Skelly, Wataru Suda, Yuki Sugiura, Seiko Narushima, et al. 2019. “A defined commensal consortium elicits CD8 T cells and anti-cancer immunity.” *Nature* 565: 600−5. <https://doi.org/10.1038/s41586-019-0878-z>

27. Zhang, Qianpeng, Yanqiu Wu, Jing Wang, Guojun Wu, Wenmin Long, Zhengsheng Xue, Linghua Wang, et al. 2016. “Accelerated dysbiosis of gut microbiota during aggravation of DSS-induced colitis by a butyrate-producing bacterium.” *Scientific Reports* 6: 27572. <https://doi.org/10.1038/srep27572>

28. Moore, W. E. C., J. L. Johnson, L. V. Holdeman. 1976. “Emendation of *Bacteroidaceae* and *Butyrivibrio* and descriptions of *Desulfomonas* gen. nov. and ten new species in the genera *Desulfomonas*, *Butyrivibrio*, *Eubacterium*, *Clostridium*, and *Ruminococcus*.” *International Journal of Systematic Bacteriology* 26: 238−52. <https://doi.org/10.1099/00207713-26-2-238>

29. Bui, Thi Phuong Nam, Willem M. de Vos, Caroline M. Plugge. 2014. “*Anaerostipes rhamnosivorans* sp. nov., a human intestinal, butyrate-forming bacterium.” *International Journal of Systematic and Evolutionary Microbiology* 64: 787−93. <https://doi.org/10.1099/ijs.0.055061-0>

30. Choi, Seung-Hyeon, Ji-Sun Kim, Jam-Eon Park, Keun Chul Lee, Mi Kyung Eom, Byeong Seob Oh, Seung Yeob Yu, et al. 2019. “*Anaerotignum faecicola* sp. nov., isolated from human faeces.” *Journal of Microbiology* 57: 1073−8. <https://doi.org/10.1007/s12275-019-9268-3>

31. Ueki, Atsuko, Kazushi Goto, Yoshimi Ohtaki, Nobuo Kaku, Katsuji Ueki. 2017. “Description of *Anaerotignum aminivorans* gen. nov., sp. nov., a strictly anaerobic, amino-acid-decomposing bacterium isolated from a methanogenic reactor, and reclassification of *Clostridium propionicum*, *Clostridium neopropionicum* and *Clostridium lactatifermentans* as species of the genus *Anaerotignum*.” *International Journal of Systematic and Evolutionary Microbiology* 67: 4146−53. <https://doi.org/10.1099/ijsem.0.002268>

32. Medvecky, Matej, Darina Cejkova, Ondrej Polansky, Daniela Karasova, Tereza Kubasova, Alois Cizek, Ivan Rychlik. 2018. “Whole genome sequencing and function prediction of 133 gut anaerobes isolated from chicken caecum in pure cultures.” *BMC Genomics* 19: 561. <https://doi.org/10.1186/s12864-018-4959-4>

33. Kant, Ravi, Pia Rasinkangas, Reetta Satokari, Taija E. Pietila, Airi Palva. 2015. “Genome sequence of the butyrate-producing anaerobic bacterium *Anaerostipes hadrus* PEL 85.” *Genome Announcements* 3: e00224-15. <https://doi.org/10.1128/genomeA.00224-15>

34. Allen-Vercoe, Emma, Michelle Daigneault, Aaron White, Remo Panaccione, Sylvia H. Duncan, Harry J. Flint, Lindsey O'Neal, Paul A. Lawson. 2012. “*Anaerostipes hadrus* comb. nov., a dominant species within the human colonic microbiota; reclassification of *Eubacterium hadrum* Moore et al. 1976.” *Anaerobe* 18: 523−9. <https://doi.org/10.1016/j.anaerobe.2012.09.002>

35. Beck, Matthias H., Anja Poehlein, Frank R. Bengelsdorf, Bettina Schiel-Bengelsdorf, Rolf Daniel, Peter Durre. 2016. “Draft genome sequence of the strict anaerobe *Clostridium neopropionicum* X4 (DSM 3847^T^).” *Genome Announcements* 4: e00209-16. <https://doi.org/10.1128/genomeA.00209-16>

36. Samain, E., G. Albagnac, H. C. Dubourguier, J. P. Touzel. 1982. “Characterization of a new propionic acid bacterium that ferments ethanol and displays a growth factor-dependent association with a Gram-negative homoacetogen.” *FEMS Microbiology Letters* 15: 69−74. <https://doi.org/10.1111/j.1574-6968.1982.tb00040.x>

37. Cardon, B. P., H. A. Barker. 1946. “Two new amino-acid-fermenting bacteria, *Clostridium propionicum* and *Diplococcus glycinophilus*.” *Journal of Bacteriology* 52: 629−34. <https://doi.org/10.1128/jb.52.6.629-634.1946>

38. Bessis, S., T. Amadou, G. Dubourg, D. Raoult, P. -E. Fournier. 2016. “"*Bariatricus massiliensis*" as a new bacterial species from human gut microbiota.” *New Microbes and New Infections* 12: 54−5. <https://doi.org/10.1016/j.nmni.2016.04.003>

39. Paek, Jayoung, Yeseul Shin, Joong-Ki Kook, Young-Hyo Chang. 2019. “*Blautia argi* sp. nov., a new anaerobic bacterium isolated from dog faeces.” *International Journal of Systematic and Evolutionary Microbiology* 69: 33−8. <https://doi.org/10.1099/ijsem.0.002981>

40. Dicks, Jo, Mohammed-Abbas Fazal, Karen Oliver, Nicholas E. Grayson, Jake D. Turnbull, Evangeline Bane, Edward Burnett, et al. 2023. “NCTC3000: a century of bacterial strain collecting leads to a rich genomic data resource.” *Microbial Genomics* 9: mgen000976. <https://doi.org/10.1099/mgen.0.000976>

41. Liu, Chengxu, Sydney M. Finegold, Yuli Song, Paul A. Lawson. 2008. “Reclassification of *Clostridium coccoides*, *Ruminococcus hansenii*, *Ruminococcus hydrogenotrophicus*, *Ruminococcus luti*, *Ruminococcus productus* and *Ruminococcus schinkii* as *Blautia coccoides* gen. nov., comb. nov., *Blautia hansenii* comb. nov., *Blautia hydrogenotrophica* comb. nov., *Blautia luti* comb. nov., *Blautia producta* comb. nov., *Blautia schinkii* comb. nov. and description of *Blautia wexlerae* sp. nov., isolated from human faeces.” *International Journal of Systematic and Evolutionary Microbiology* 58: 1896−902. <https://doi.org/10.1099/ijs.0.65208-0>

42. Kaneuchi, Choji, Yoshimi Benno, Tomotari Mitsuoka. 1976. “*Clostridium coccoides*, a new species from the feces of mice.” *International Journal of Systematic Bacteriology* 26: 482−6. <https://doi.org/10.1099/00207713-26-4-482>

43. Garzetti, Debora, Sandrine Brugiroux, Boyke Bunk, Rudiger Pukall, Kathy D. McCoy, Andrew J. Macpherson, Barbel Stecher. 2017. “High-quality whole-genome sequences of the Oligo-Mouse-Microbiota bacterial community.” *Genome Announcements* 5: e00758-17. <https://doi.org/10.1128/genomeA.00758-17>

44. Kim, Ji-Sun, Jam-Eon Park, Keun Chul Lee, Seung-Hyeon Choi, Byeong Seob Oh, Seung Yeob Yu, Mi Kyung Eom, et al. 2020. “*Blautia faecicola* sp. nov., isolated from faeces from a healthy human.” *International Journal of Systematic and Evolutionary Microbiology* 70: 2059−65. <https://doi.org/10.1099/ijsem.0.004015>

45. Park, Seong-Kyu, Min-Soo Kim, Jin-Woo Bae. 2013. “*Blautia faecis* sp. nov., isolated from human faeces.” *International Journal of Systematic and Evolutionary Microbiology* 63: 599−603. <https://doi.org/10.1099/ijs.0.036541-0>

46. Ezaki, Takayuki, Na Li, Yasuhiro Hashimoto, Hiroaki Miura, Hiroaki Yamamoto. 1994. “16S ribosomal DNA sequences of anaerobic cocci and proposal of *Ruminococcus hansenii* comb. nov. and *Ruminococcus productus* comb. nov.” *International Journal of Systematic Bacteriology* 44: 130−6. <https://doi.org/10.1099/00207713-44-1-130>

47. Shin, Na-Ri, Woorim Kang, Euon Jung Tak, Dong-Wook Hyun, Pil Soo Kim, Hyun Sik Kim, June-Young Lee, Hojun Sung, Tae Woong Whon, Jin-Woo Bae. 2018. “*Blautia hominis* sp. nov., isolated from human faeces.” *International Journal of Systematic and Evolutionary Microbiology* 68: 1059−64. <https://doi.org/10.1099/ijsem.0.002623>

48. Integrative, H. M. P. Research Network Consortium. 2019. “The Integrative Human Microbiome Project.” *Nature* 569: 641−8. <https://doi.org/10.1038/s41586-019-1238-8>

49. Bernalier, A., Anne Willems, Marion Leclerc, Violaine Rochet, Matthew D. Collins. 1996. “*Ruminococcus hydrogenotrophicus* sp. nov., a new H2/CO2-utilizing acetogenic bacterium isolated from human feces.” *Archives of Microbiology* 166: 176−83. <https://doi.org/10.1007/s002030050373>

50. Raman, Arjun S., Jeanette L. Gehrig, Siddarth Venkatesh, Hao-Wei Chang, Matthew C. Hibberd, Sathish Subramanian, Gagandeep Kang, et al. 2019. “A sparse covarying unit that describes healthy and impaired human gut microbiota development.” *Science* 365: eaau4735. <https://doi.org/10.1126/science.aau4735>

51. Gehrig, Jeanette L., Siddarth Venkatesh, Hao-Wei Chang, Matthew C. Hibberd, Vanderlene L. Kung, Jiye Cheng, Robert Y. Chen, et al. 2019. “Effects of microbiota-directed foods in gnotobiotic animals and undernourished children.” *Science* 365: eaau4732. <https://doi.org/10.1126/science.aau4732>

52. Simmering, Rainer, David Taras, Andreas Schwiertz, Gwenaelle Le Blay, Barbel Gruhl, Paul A. Lawson, Matrhew D. Collins, Michael Blaut. 2002. “*Ruminococcus luti* sp. nov., isolated from a human faecal sample.” *Systematic and Applied Microbiology* 25: 189−93. <https://doi.org/10.1078/0723-2020-00112>

53. Hatziioanou, Diane, Cristina Gherghisan-Filip, Gerhard Saalbach, Nikki Horn, Udo Wegmann, Sylvia H. Duncan, Harry J. Flint, Melinda J. Mayer, Arjan Narbad. 2017. “Discovery of a novel lantibiotic nisin O from *Blautia obeum* A2-162, isolated from the human gastrointestinal tract.” *Microbiology* 163: 1292−305. <https://doi.org/10.1099/mic.0.000515>

54. Ehrlich, S. Dusko. 2011. MetaHIT: The European Union Project on metagenomics of the human intestinal tract. *Metagenomics of the Human Body* Springer, 307−16. <https://doi.org/10.1007/978-1-4419-7089-3_15>

55. Jiang, Xiaofang, A. Brantley Hall, Timothy D. Arthur, Damian R. Plichta, Christian T. Covington, Mathilde Poyet, Jessica Crothers, et al. 2019. “Invertible promoters mediate bacterial phase variation, antibiotic resistance, and host adaptation in the gut.” *Science* 363: 181−7. <https://doi.org/10.1126/science.aau5238>

56. Fitzgerald, Cormac Brian, Andrey N. Shkoporov, Thomas D. S. Sutton, Andrei V. Chaplin, Vimalkumar Velayudhan, R. Paul Ross, Colin Hill. 2018. “Comparative analysis of *Faecalibacterium prausnitzii* genomes shows a high level of genome plasticity and warrants separation into new species-level taxa.” *BMC Genomics* 19: 931. <https://doi.org/10.1186/s12864-018-5313-6>

57. Kim, Mihyang, Nayoung Kim, Jaehong Han. 2014. “Metabolism of *Kaempferia parviflora* polymethoxyflavones by human intestinal bacterium *Bautia* sp. MRG-PMF1.” *Journal of Agricultural and Food Chemistry* 62: 12377−83. <https://doi.org/10.1021/jf504074n>

58. Lawson, Paul A., Sydney M. Finegold. 2015. “Reclassification of *Ruminococcus obeum* as *Blautia obeum* comb. nov.” *International Journal of Systematic and Evolutionary Microbiology* 65: 789−93. <https://doi.org/10.1099/ijs.0.000015>

59. Kim, Sohn G., Simone Becattini, Thomas U. Moody, Pavel V. Shliaha, Eric R. Littmann, Ruth Seok, Mergim Gjonbalaj, et al. 2019. “Microbiota-derived lantibiotic restores resistance against vancomycin-resistant *Enterococcus*.” *Nature* 572: 665−9. <https://doi.org/10.1038/s41586-019-1501-z>

60. Rettedal, Elizabeth A., Heidi Gumpert, Morten O. A. Sommer. 2014. “Cultivation-based multiplex phenotyping of human gut microbiota allows targeted recovery of previously uncultured bacteria.” *Nature Communications* 5: 4714. <https://doi.org/10.1038/ncomms5714>

61. Seshadri, Rekha, Sinead C. Leahy, Graeme T. Attwood, Koon Hoong Teh, Suzanne C. Lambie, Adrian L. Cookson, Emiley A. Eloe-Fadrosh, et al. 2018. “Cultivation and sequencing of rumen microbiome members from the Hungate1000 Collection.” *Nature Biotechnology* 36: 359−67. <https://doi.org/10.1038/nbt.4110>

62. Rieu-Lesme, F., B. Morvan, M. D. Collins, G. Fonty, A. Willems. 1996. “A new H2/CO2-using acetogenic bacterium from the rumen: description of *Ruminococcus schinkii* sp. nov.” *FEMS Microbiology Letters* 140: 281−6. <https://doi.org/10.1016/0378-1097(96)00195-4>

63. Mukherjee, Supratim, Rekha Seshadri, Neha J. Varghese, Emiley A. Eloe-Fadrosh, Jan P. Meier-Kolthoff, Markus Goker, R. Cameron Coates, et al. 2017. “1,003 reference genomes of bacterial and archaeal isolates expand coverage of the tree of life.” *Nature Biotechnology* 35: 676−83. <https://doi.org/10.1038/nbt.3886>

64. Rodriguez Hernaez, Javier, Maria Esperanza Ceron Cucchi, Silvio Cravero, Maria Carolina Martinez, Sergio Gonzalez, Andrea Puebla, Joaquin Dopazo, Marisa Farber, Norma Paniego, Maximo Rivarola. 2018. “The first complete genomic structure of *Butyrivibrio fibrisolvens* and its chromid.” *Microbial Genomics* 4: e000216. <https://doi.org/10.1099/mgen.0.000216>

65. Rumney, Corinne J., Sylvia H. Duncan, C. Henderson, C. S. Stewart. 1995. “Isolation and characteristics of a wheatbran-degrading *Butyrivibrio* from human faeces.” *Letters in Applied Microbiology* 20: 232−6. <https://doi.org/10.1111/j.1472-765x.1995.tb00435.x>

66. Bryant, Marvin P., Nola Small. 1956. “The anaerobic monotrichous butyric acid-producing curved rod-shaped bacteria of the rumen.” *Journal of Bacteriology* 72: 16−21. <https://doi.org/10.1128/jb.72.1.16-21.1956>

67. Palevich, Nikola, William J. Kelly, Siva Ganesh, Jasna Rakonjac, Graeme T. Attwood. 2019. “*Butyrivibrio hungatei* MB2003 competes effectively for soluble sugars released by *Butyrivibrio proteoclasticus* B316^T^ during growth on xylan or pectin.” *Applied and Environmental Microbiology* 85: e02056-18. <https://doi.org/10.1128/AEM.02056-18>

68. Palevich, Nikola, William J. Kelly, Sinead C. Leahy, Stuart Denman, Eric Altermann, Jasna Rakonjac, Graeme T. Attwood. 2019. “Comparative genomics of *Rumen Butyrivibrio* spp. uncovers a continuum of polysaccharide-degrading capabilities.” *Applied and Environmental Microbiology* 86: e01993-19. <https://doi.org/10.1128/AEM.01993-19>

69. Palevich, Nikola, William J. Kelly, Sinead C. Leahy, Eric Altermann, Jasna Rakonjac, Graeme T. Attwood. 2017. “The complete genome sequence of the rumen bacterium *Butyrivibrio hungatei* MB2003.” *Standards in Genomic Sciences* 12: 72. <https://doi.org/10.1186/s40793-017-0285-8>

70. Kopecny, Jan, Masa Zorec, Jakub Mrazek, Yasuo Kobayashi, Romana Marinsek-Logar. 2003. “*Butyrivibrio hungatei* sp. nov. and *Pseudobutyrivibrio xylanivorans* sp. nov., butyrate-producing bacteria from the rumen.” *International Journal of Systematic and Evolutionary Microbiology* 53: 201−9. <https://doi.org/10.1099/ijs.0.02345-0>

71. Kelly, William J., Sinead C. Leahy, Eric Altermann, Carl J. Yeoman, Jonathan C. Dunne, Zhanhao Kong, Diana M. Pacheco, et al. 2010. “The glycobiome of the rumen bacterium *Butyrivibrio proteoclasticus* B316^T^ highlights adaptation to a polysaccharide-rich environment.” *PloS One* 5: e11942. <https://doi.org/10.1371/journal.pone.0011942>

72. Braune, Annett, Michael Blaut. 2018. “*Catenibacillus scindens* gen. nov., sp. nov., a C-deglycosylating human intestinal representative of the Lachnospiraceae.” *International Journal of Systematic and Evolutionary Microbiology* 68: 3356−61. <https://doi.org/10.1099/ijsem.0.003001>

73. Moore, Lillian V. H., W. E. C. Moore. 1994. “*Oribaculum catoniae* gen. nov., sp. nov.; *Catonella morbi* gen. nov., sp. nov.; *Hallella seregens* gen. nov., sp. nov.; *Johnsonella ignava* gen. nov., sp. nov.; and *Dialister pneumosintes* gen. nov., comb. nov., nom. rev., Anaerobic gram-negative bacilli from the human gingival crevice.” *International Journal of Systematic Bacteriology* 44: 187−92. <https://doi.org/10.1099/00207713-44-2-187>

74. Cai, Shichun, Xiuzhu Dong. 2010. “*Cellulosilyticum ruminicola* gen. nov., sp. nov., isolated from the rumen of yak, and reclassification of *Clostridium lentocellum* as *Cellulosilyticum lentocellum* comb. nov.” *International Journal of Systematic and Evolutionary Microbiology* 60: 845−9. <https://doi.org/10.1099/ijs.0.014712-0>

75. Murray, William D., Lisa Hofmann, Nancy L. Campbell, Robert H. Madden. 1986. “*Clostridium lentocellum* sp. nov., a cellulolytic species from river sediment containing paper-mill waste.” *Systematic and Applied Microbiology* 8: 181−4. <https://doi.org/10.1016/s0723-2020(86)80074-1>

76. Atarashi, Koji, Takeshi Tanoue, Kenshiro Oshima, Wataru Suda, Yuji Nagano, Hiroyoshi Nishikawa, Shinji Fukuda, et al. 2013. “T_reg_ induction by a rationally selected mixture of Clostridia strains from the human microbiota.” *Nature* 500: 232−6. <https://doi.org/10.1038/nature12331>

77. Reichardt, Nicole, Sylvia H. Duncan, Pauline Young, Alvaro Belenguer, Carol McWilliam Leitch, Karen P. Scott, Harry J. Flint, Petra Louis. 2014. “Phylogenetic distribution of three pathways for propionate production within the human gut microbiota.” *The ISME Journal* 8: 1323−35. <https://doi.org/10.1038/ismej.2014.14>

78. Zou, Ling, Peter Spanogiannopoulos, Lindsey M. Pieper, Huan-Chieh Chien, Wenlong Cai, Natalia Khuri, Joshua Pottel, et al. 2020. “Bacterial metabolism rescues the inhibition of intestinal drug absorption by food and drug additives.” *Proceedings of the National Academy of Sciences of the United States of America* 117: 16009−18. <https://doi.org/10.1073/pnas.1920483117>

79. Sichtig, Heike, Timothy Minogue, Yi Yan, Christopher Stefan, Adrienne Hall, Luke Tallon, Lisa Sadzewicz, et al. 2019. “FDA-ARGOS is a database with public quality-controlled reference genomes for diagnostic use and regulatory science.” *Nature Communications* 10: 3313. <https://doi.org/10.1038/s41467-019-11306-6>

80. Taras, David, Rainer Simmering, Matthew D. Collins, Paul A. Lawson, Michael Blaut. 2002. “Reclassification of *Eubacterium formicigenerans* Holdeman and Moore 1974 as *Dorea formicigenerans* gen. nov., comb. nov., and description of *Dorea longicatena* sp. nov., isolated from human faeces.” *International Journal of Systematic and Evolutionary Microbiology* 52: 423−8. <https://doi.org/10.1099/00207713-52-2-423>

81. Huang, Yiming, Ravi U. Sheth, Andrew Kaufman, Harris H. Wang. 2020. “Scalable and cost-effective ribonuclease-based rRNA depletion for transcriptomics.” *Nucleic Acids Research* 48: e20. <https://doi.org/10.1093/nar/gkz1169>

82. Takakura, T., R. Francis, H. Anani, S. Naud, D. Raoult, J. Y. Bou Khalil. 2019. “*Dorea phocaeensis* sp. nov., a new bacterium isolated from the stool of a healthy 29-year-old male.” *New Microbes and New Infections* 32: 100600. <https://doi.org/10.1016/j.nmni.2019.100600>

83. Bilen, M., M. D. Mbogning Founkou, F. Cadoret, G. Dubourg, Z. Daoud, D. Raoult. 2018. “*Sanguibacter massiliensis* sp. nov., *Actinomyces minihominis* sp. nov., *Clostridium minihomine* sp. nov., *Neobittarella massiliensis* gen. nov. and *Miniphocibacter massiliensis* gen. nov., new bacterial species isolated by culturomics from human stool samples.” *New Microbes and New Infections* 24: 21−5. <https://doi.org/10.1016/j.nmni.2018.03.002>

84. Alou, M. T., P. -E. Fournier, D. Raoult. 2016. “"*Africanella massiliensis*," a new bacterial genus isolated from human gut microbiota.” *New Microbes and New Infections* 12: 99−100. <https://doi.org/10.1016/j.nmni.2016.05.008>

85. Togo, Amadou H., Awa Diop, Matthieu Million, Marie Maraninchi, Jean-Christophe Lagier, Catherine Robert, Fabrizio Di Pinto, Didier Raoult, Pierre-Edouard Fournier, Fadi Bittar. 2018. “Draft genome and description of *Eisenbergiella massiliensis* strain AT11^T^: a new species isolated from human feces after bariatric surgery.” *Current Microbiology* 75: 1274−81. <https://doi.org/10.1007/s00284-018-1520-2>

86. Bernard, Kathryn, Tamara Burdz, Deborah Wiebe, Brittany M. Balcewich, Tina Zimmerman, Philippe Lagace-Wiens, Linda M. N. Hoang, Anne-Marie Bernier. 2017. “Characterization of isolates of *Eisenbergiella tayi*, a strictly anaerobic Gram-stain variable bacillus recovered from human clinical materials in Canada.” *Anaerobe* 44: 128−32. <https://doi.org/10.1016/j.anaerobe.2017.03.005>

87. Amir, Itay, Philippe Bouvet, Christine Legeay, Uri Gophna, Abraham Weinberger. 2014. “*Eisenbergiella tayi* gen. nov., sp. nov., isolated from human blood.” *International Journal of Systematic and Evolutionary Microbiology* 64: 907−14. <https://doi.org/10.1099/ijs.0.057331-0>

88. Lagier, Jean-Christophe, Saber Khelaifia, Maryam Tidjani Alou, Sokhna Ndongo, Niokhor Dione, Perrine Hugon, Aurelia Caputo, et al. 2016. “Culture of previously uncultured members of the human gut microbiota by culturomics.” *Nature Microbiology* 1: 16203. <https://doi.org/10.1038/nmicrobiol.2016.203>

89. Dubourg, Gregory, Sophie Baron, Frederic Cadoret, Carine Couderc, Pierre-Edouard Fournier, Jean-Christophe Lagier, Didier Raoult. 2018. “From culturomics to clinical microbiology and forward.” *Emerging Infectious Diseases* 24: 1683−90. <https://doi.org/10.3201/eid2409.170995>

90. Haas, Kelly N., Jeffrey L. Blanchard. 2020. “Reclassification of the *Clostridium clostridioforme* and *Clostridium sphenoides* clades as *Enterocloster* gen. nov. and *Lacrimispora* gen. nov., including reclassification of 15 taxa.” *International Journal of Systematic and Evolutionary Microbiology* 70: 23−34. <https://doi.org/10.1099/ijsem.0.003698>

91. Dehoux, Pierre, Jean Christophe Marvaud, Amr Abouelleil, Ashlee M. Earl, Thierry Lambert, Catherine Dauga. 2016. “Comparative genomics of *Clostridium bolteae* and *Clostridium clostridioforme* reveals species-specific genomic properties and numerous putative antibiotic resistance determinants.” *BMC Genomics* 17: 819. <https://doi.org/10.1186/s12864-016-3152-x>

92. Liu, Chang, Nan Zhou, Meng-Xuan Du, Yu-Tong Sun, Kai Wang, Yu-Jing Wang, Dan-Hua Li, et al. 2020. “The Mouse Gut Microbial Biobank expands the coverage of cultured bacteria.” *Nature Communications* 11: 79. <https://doi.org/10.1038/s41467-019-13836-5>

93. Sakamoto, Mitsuo, Takao Iino, Moriya Ohkuma. 2017. “*Faecalimonas umbilicata* gen. nov., sp. nov., isolated from human faeces, and reclassification of *Eubacterium contortum*, *Eubacterium fissicatena* and *Clostridium oroticum* as *Faecalicatena contorta* gen. nov., comb. nov., *Faecalicatena fissicatena* comb. nov. and *Faecalicatena orotica* comb. nov.” *International Journal of Systematic and Evolutionary Microbiology* 67: 1219−27. <https://doi.org/10.1099/ijsem.0.001790>

94. Koeck, Daniela E., Irena Maus, Daniel Wibberg, Anika Winkler, Vladimir V. Zverlov, Wolfgang Liebl, Alfred Puhler, Wolfgang H. Schwarz, Andreas Schluter. 2015. “Draft genome sequence of *Herbinix hemicellulosilytica* T3/55^T^, a new thermophilic cellulose degrading bacterium isolated from a thermophilic biogas reactor.” *Journal of Biotechnology* 214: 59−60. <https://doi.org/10.1016/j.jbiotec.2015.07.022>

95. Koeck, Daniela E., Wolfgang Ludwig, Gerhard Wanner, Vladimir V. Zverlov, Wolfgang Liebl, Wolfgang H. Schwarz. 2015. “*Herbinix hemicellulosilytica* gen. nov., sp. nov., a thermophilic cellulose-degrading bacterium isolated from a thermophilic biogas reactor.” *International Journal of Systematic and Evolutionary Microbiology* 65: 2365−71. <https://doi.org/10.1099/ijs.0.000264>

96. Beri, Dhananjay, William S. York, Lee R. Lynd, Maria J. Pena, Christopher D. Herring. 2020. “Development of a thermophilic coculture for corn fiber conversion to ethanol.” *Nature Communications* 11: 1937. <https://doi.org/10.1038/s41467-020-15704-z>

97. Koeck, Daniela E., Sarah Hahnke, Vladimir V. Zverlov. 2016. “*Herbinix luporum* sp. nov., a thermophilic cellulose-degrading bacterium isolated from a thermophilic biogas reactor.” *International Journal of Systematic and Evolutionary Microbiology* 66: 4132−7. <https://doi.org/10.1099/ijsem.0.001324>

98. Whitehead, Terence R., Michael A. Cotta, Matthew D. Collins, Paul A. Lawson. 2004. “*Hespellia stercorisuis* gen. nov., sp. nov. and *Hespellia porcina* sp. nov., isolated from swine manure storage pits.” *International Journal of Systematic and Evolutionary Microbiology* 54: 241−5. <https://doi.org/10.1099/ijs.0.02719-0>

99. Haas, Kelly Nicole, Jeffrey L. Blanchard. 2017. “*Kineothrix alysoides*, gen. nov., sp. nov., a saccharolytic butyrate-producer within the family Lachnospiraceae.” *International Journal of Systematic and Evolutionary Microbiology* 67: 402−10. <https://doi.org/10.1099/ijsem.0.001643>

100. Hedberg, Maria E., Edward R. B. Moore, Liselott Svensson-Stadler, Per Horstedt, Vladimir Baranov, Olle Hernell, Sun Nyunt Wai, Sten Hammarstrom, Marie-Louise Hammarstrom. 2012. “*Lachnoanaerobaculum* gen. nov., a new genus in the Lachnospiraceae: characterization of *Lachnoanaerobaculum umeaense* gen. nov., sp. nov., isolated from the human small intestine, and *Lachnoanaerobaculum orale* sp. nov., isolated from saliva, and reclassification of *Eubacterium saburreum* (Prevot 1966) Holdeman and Moore 1970 as *Lachnoanaerobaculum saburreum* comb. nov.” *International Journal of Systematic and Evolutionary Microbiology* 62: 2685−90. <https://doi.org/10.1099/ijs.0.033613-0>

101. Sizova, M. V., T. Hohmann, A. Hazen, B. J. Paster, S. R. Halem, C. M. Murphy, N. S. Panikov, S. S. Epstein. 2012. “New approaches for isolation of previously uncultivated oral bacteria.” *Applied and Environmental Microbiology* 78: 194−203. <https://doi.org/10.1128/AEM.06813-11>

102. Whitford, M. F., L. J. Yanke, R. J. Forster, R. M. Teather. 2001. “*Lachnobacterium bovis* gen. nov., sp. nov., a novel bacterium isolated from the rumen and faeces of cattle.” *International Journal of Systematic and Evolutionary Microbiology* 51: 1977−81. <https://doi.org/10.1099/00207713-51-6-1977>

103. Traore, S. I., E. I. Azhar, M. Yasir, F. Bibi, P. -E. Fournier, A. A. Jiman-Fatani, J. Delerce, F. Cadoret, J. -C. Lagier, D. Raoult. 2017. “Description of '*Blautia phocaeensis*' sp. nov. and '*Lachnoclostridium edouardi*' sp. nov., isolated from healthy fresh stools of Saudi Arabia Bedouins by culturomics.” *New Microbes and New Infections* 19: 129−31. <https://doi.org/10.1016/j.nmni.2017.05.017>

104. Brahimi, S., F. Cadoret, P. -E. Fournier, V. Moal, D. Raoult. 2017. “'*Lachnoclostridium urinimassiliense*' sp. nov. and '*Lachnoclostridium phocaeense*' sp. nov., two new bacterial species isolated from human urine after kidney transplantation.” *New Microbes and New Infections* 16: 73−5. <https://doi.org/10.1016/j.nmni.2017.01.008>

105. Dandachi, I., H. Anani, L. Hadjadj, S. Brahimi, J. -C. Lagier, Z. Daoud, J. -M. Rolain. 2021. “Genome analysis of *Lachnoclostridium phocaeense* isolated from a patient after kidney transplantation in Marseille.” *New Microbes and New Infections* 41: 100863. <https://doi.org/10.1016/j.nmni.2021.100863>

106. Haas, Kelly N. 2016. “Expansion of and reclassification within the family Lachnospiraceae.” *Doctoral Dissertations* 843. <https://doi.org/10.7275/9055799.0>

107. Warnick, Thomas A., Barbara A. Methe, Susan B. Leschine. 2002. “*Clostridium phytofermentans* sp. nov., a cellulolytic mesophile from forest soil.” *International Journal of Systematic and Evolutionary Microbiology* 52: 1155−60. <https://doi.org/10.1099/00207713-52-4-1155>

108. Mahowald, Michael A., Federico E. Rey, Henning Seedorf, Peter J. Turnbaugh, Robert S. Fulton, Aye Wollam, Neha Shah, et al. 2009. “Characterizing a model human gut microbiota composed of members of its two dominant bacterial phyla.” *Proceedings of the National Academy of Sciences of the United States of America* 106: 5859−64. <https://doi.org/10.1073/pnas.0901529106>

109. Garcia-Lopez, Marina, Jan P. Meier-Kolthoff, Brian J. Tindall, Sabine Gronow, Tanja Woyke, Nikos C. Kyrpides, Richard L. Hahnke, Markus Goker. 2019. “Analysis of 1,000 type-strain genomes improves taxonomic classification of bacteroidetes.” *Frontiers in Microbiology* 10: 2083. <https://doi.org/10.3389/fmicb.2019.02083>

110. Huang, Chien-Hsun, Jong-Shian Liou, Chun-Lin Wang, Lina Huang. 2018. “Draft genome sequence of *Clostridium* sp. strain chh4-2 isolated from human feces.” *Genome Announcements* 6: e00070-18. <https://doi.org/10.1128/genomeA.00070-18>

111. Jarzembowska, Monika, Diana Z. Sousa, Florian Beyer, Arie Zwijnenburg, Caroline M. Plugge, Alfons J. M. Stams. 2016. “*Lachnotalea glycerini* gen. nov., sp. nov., an anaerobe isolated from a nanofiltration unit treating anoxic groundwater.” *International Journal of Systematic and Evolutionary Microbiology* 66: 774−9. <https://doi.org/10.1099/ijsem.0.000791>

112. Maheux, Andree F., Dominique K. Boudreau, Eve Berube, Maurice Boissinot, Frederic Raymond, Stephanie Brodeur, Jacques Corbeil, Sandra Isabel, Rabeea F. Omar, Michel G. Bergeron. 2017. “Draft genome sequence of a sporulating and motile strain of *Lachnotalea glycerini* isolated from water in Quebec City, Canada.” *Genome Announcements* 5: e01059-17. <https://doi.org/10.1128/genomeA.01059-17>

113. Broda, D. M., D. J. Saul, R. G. Bell, D. R. Musgrave. 2000. “*Clostridium algidixylanolyticum* sp. nov., a psychrotolerant, xylan-degrading, spore-forming bacterium.” *International Journal of Systematic and Evolutionary Microbiology* 50 Pt 2: 623−31. <https://doi.org/10.1099/00207713-50-2-623>

114. Sleat, Robert, Robert A. Mah, Ralph Robinson. 1984. “Isolation and characterization of an anaerobic, cellulolytic bacterium, *Clostridium cellulovorans* sp. nov.” *Applied and Environmental Microbiology* 48: 88−93. <https://doi.org/10.1128/aem.48.1.88-93.1984>

115. Wolin, Meyer J., Terry L. Miller, Paul A. Lawson. 2008. “Proposal to replace the illegitimate genus name *Bryantella* Wolin *et al.* 2004^VP^ with the genus name *Marvinbryantia* gen. nov. and to replace the illegitimate combination *Bryantella formatexigens* Wolin *et al.* 2004^VP^ with *Marvinbryantia formatexigens* comb. nov.” *International Journal of Systematic and Evolutionary Microbiology* 58: 742−4. <https://doi.org/10.1099/ijs.0.65850-0>

116. Wolin, Meyer J., Terry L. Miller, Matthew D. Collins, Paul A. Lawson. 2003. “Formate-dependent growth and homoacetogenic fermentation by a bacterium from human feces: description of *Bryantella formatexigens* gen. nov., sp. nov.” *Applied and Environmental Microbiology* 69: 6321−6. <https://doi.org/10.1128/AEM.69.10.6321-6326.2003>

117. Togo, Amadou Hamidou, Awa Diop, Fadi Bittar, Marie Maraninchi, Rene Valero, Nicholas Armstrong, Gregory Dubourg, et al. 2018. “Description of *Mediterraneibacter massiliensis*, gen. nov., sp. nov., a new genus isolated from the gut microbiota of an obese patient and reclassification of *Ruminococcus faecis*, *Ruminococcus lactaris*, *Ruminococcus torques*, *Ruminococcus gnavus* and *Clostridium glycyrrhizinilyticum* as *Mediterraneibacter faecis* comb. nov., *Mediterraneibacter lactaris* comb. nov., *Mediterraneibacter torques* comb. nov., *Mediterraneibacter gnavus* comb. nov. and *Mediterraneibacter glycyrrhizinilyticus* comb. nov.” *Antonie Van Leeuwenhoek* 111: 2107−28. <https://doi.org/10.1007/s10482-018-1104-y>

118. Kim, Min-Soo, Seong Woon Roh, Jin-Woo Bae. 2011. “*Ruminococcus faecis* sp. nov., isolated from human faeces.” *Journal of Microbiology* 49: 487−91. <https://doi.org/10.1007/s12275-011-0505-7>

119. Sakuma, Keita, Maki Kitahara, Ryoko Kibe, Mitsuo Sakamoto, Yoshimi Benno. 2006. “*Clostridium glycyrrhizinilyticum* sp. nov., a glycyrrhizin-hydrolysing bacterium isolated from human faeces.” *Microbiology and immunology* 50: 481−5. <https://doi.org/10.1111/j.1348-0421.2006.tb03818.x>

120. Togo, A. H., M. Maraninchi, F. Bittar, D. Raoult, M. Million. 2016. “"*Ruminococcus phoceensis*," a new species identified from human stool from an obese patient before bariatric surgery.” *New Microbes and New Infections* 14: 67−8. <https://doi.org/10.1016/j.nmni.2016.09.004>

121. Sakamoto, Mitsuo, Nao Ikeyama, Masahiro Yuki, Moriya Ohkuma. 2018. “Draft genome sequence of *Faecalimonas umbilicata* JCM 30896^T^, an acetate-producing bacterium isolated from human feces.” *Microbiology Resource Announcements* 7: e01091-18. <https://doi.org/10.1128/MRA.01091-18>

122. Sizova, Maria V., Paul A. Muller, David Stancyk, Nicolai S. Panikov, Manolis Mandalakis, Amanda Hazen, Tine Hohmann, et al. 2014. “*Oribacterium parvum* sp. nov. and *Oribacterium asaccharolyticum* sp. nov., obligately anaerobic bacteria from the human oral cavity, and emended description of the genus *Oribacterium*.” *International Journal of Systematic and Evolutionary Microbiology* 64: 2642−9. <https://doi.org/10.1099/ijs.0.060988-0>

123. Kalyani, Dayanand C., Tom Reichenbach, Henrik Aspeborg, Christina Divne. 2021. “A homodimeric bacterial exo-beta-1,3-glucanase derived from moose rumen microbiome shows a structural framework similar to yeast exo-beta-1,3-glucanases.” *Enzyme and Microbial Technology* 143: 109723. <https://doi.org/10.1016/j.enzmictec.2020.109723>

124. Peng, Xuefeng, St Elmo Wilken, Thomas S. Lankiewicz, Sean P. Gilmore, Jennifer L. Brown, John K. Henske, Candice L. Swift, et al. 2021. “Genomic and functional analyses of fungal and bacterial consortia that enable lignocellulose breakdown in goat gut microbiomes.” *Nature Microbiology* 6: 499−511. <https://doi.org/10.1038/s41564-020-00861-0>

125. Pidcock, Sara E., Timofey Skvortsov, Fernanda G. Santos, Stephen J. Courtney, Karen Sui-Ting, Christopher J. Creevey, Sharon A. Huws. 2021. “Phylogenetic systematics of *Butyrivibrio* and *Pseudobutyrivibrio* genomes illustrate vast taxonomic diversity, open genomes and an abundance of carbohydrate-active enzyme family isoforms.” *Microbial Genomics* 7: 000638. <https://doi.org/10.1099/mgen.0.000638>

126. Braasch, Jeanna L., Carly N. Lapin, Scot E. Dowd, Richard W. McLaughlin. 2015. “Draft genome sequence of *Robinsoniella peoriensis* strain WTD, isolated from the fecal material of a wood turtle.” *Genome Announcements* 3: e01444-14. <https://doi.org/10.1128/genomeA.01444-14>

127. Schrottner, Percy, Kathleen Hartwich, Boyke Bunk, Isabel Schober, Sina Helbig, Wolfram W. Rudolph, Florian Gunzer. 2019. “Detection of *Robinsoniella peoriensis* in multiple bone samples of a trauma patient.” *Anaerobe* 59: 14−8. <https://doi.org/10.1016/j.anaerobe.2019.05.001>

128. Hatziioanou, Diane, Melinda J. Mayer, Sylvia H. Duncan, Harry J. Flint, Arjan Narbad. 2013. “A representative of the dominant human colonic Firmicutes, *Roseburia faecis* M72/1, forms a novel bacteriocin-like substance.” *Anaerobe* 23: 5−8. <https://doi.org/10.1016/j.anaerobe.2013.07.006>

129. Duncan, Sylvia H., Rustam I. Aminov, Karen P. Scott, Petra Louis, Thaddeus B. Stanton, Harry J. Flint. 2006. “Proposal of *Roseburia faecis* sp. nov., *Roseburia hominis* sp. nov. and *Roseburia inulinivorans* sp. nov., based on isolates from human faeces.” *International Journal of Systematic and Evolutionary Microbiology* 56: 2437−41. <https://doi.org/10.1099/ijs.0.64098-0>

130. Travis, Anthony J., Denise Kelly, Harry J. Flint, Rustam I. Aminov. 2015. “Complete genome sequence of the human gut symbiont *Roseburia hominis*.” *Genome Announcements* 3: e01286-15. <https://doi.org/10.1128/genomeA.01286-15>

131. Aminov, Rustam I., Alan W. Walker, Sylvia H. Duncan, Hermie J. M. Harmsen, Gjalt W. Welling, Harry J. Flint. 2006. “Molecular diversity, cultivation, and improved detection by fluorescent in situ hybridization of a dominant group of human gut bacteria related to *Roseburia* spp. or *Eubacterium rectale*.” *Applied and environmental microbiology* 72: 6371−6. <https://doi.org/10.1128/AEM.00701-06>

132. Louis, Petra, Sylvia H. Duncan, Sheila I. McCrae, Jacqueline Millar, Michelle S. Jackson, Harry J. Flint. 2004. “Restricted distribution of the butyrate kinase pathway among butyrate-producing bacteria from the human colon.” *Journal of Bacteriology* 186: 2099−106. <https://doi.org/10.1128/JB.186.7.2099-2106.2004>

133. Soh, Melissa, Sou Miyake, Austin Lim, Yichen Ding, Henning Seedorf. 2019. “*Schaedlerella arabinosiphila* gen. nov., sp. nov., a D-arabinose-utilizing bacterium isolated from faeces of C57BL/6J mice that is a close relative of *Clostridium* species ASF 502.” *International Journal of Systematic and Evolutionary Microbiology* 69: 3616−22. <https://doi.org/10.1099/ijsem.0.003671>

134. Wannemuehler, Michael J., Ann-Marie Overstreet, Doyle V. Ward, Gregory J. Phillips. 2014. “Draft genome sequences of the altered Schaedler flora, a defined bacterial community from gnotobiotic mice.” *Genome Announcements* 2: e00287-14. <https://doi.org/10.1128/genomeA.00287-14>

135. Durand, G. A., J. -C. Lagier, S. Khelaifia, N. Armstrong, C. Robert, J. Rathored, P. -E. Fournier, D. Raoult. 2016. “*Drancourtella massiliensis* gen. nov., sp. nov. isolated from fresh healthy human faecal sample from South France.” *New Microbes and New Infections* 11: 34−42. <https://doi.org/10.1016/j.nmni.2016.02.002>

136. Rasmussen, Torben Solbeck, Theresa Streidl, Thomas C. A. Hitch, Esther Wortmann, Paulina Deptula, Michael V. W. Kofoed, Thomas Riedel, et al. 2019. “*Sporofaciens musculi* gen. nov., sp. nov., a novel bacterium isolated from the caecum of an obese mouse.” *International Journal of Systematic and Evolutionary Microbiology* 71: 004673. <https://doi.org/10.1099/ijsem.0.004673>

137. Zenner, Christian, Thomas C. A. Hitch, Thomas Riedel, Esther Wortmann, Stefan Tiede, Eva M Buhl, Birte Abt, et al. 2021. “Early-life immune system maturation in chickens using a synthetic community of cultured gut bacteria.” *mSystems* 6: e01300-20. <https://doi.org/10.1128/mSystems.01300-20>
